# Therapeutic potential and impact of nanoengineered patient‐derived mesenchymal stem cells in a murine resection and recurrence model of human glioblastoma

**DOI:** 10.1002/btm2.10675

**Published:** 2024-05-07

**Authors:** Rawan Al‐Kharboosh, Alex Bechtle, Stephany Y. Tzeng, Jiaying Zheng, Sujan Kumar Mondal, David R. Wilson, Carlos Perez‐Vega, Jordan J. Green, Alfredo Quiñones‐Hinojosa

**Affiliations:** ^1^ Department of Neurosurgery Mayo Clinic Jacksonville Florida USA; ^2^ Department of Neuroscience Mayo Clinic Graduate School Jacksonville Florida USA; ^3^ AtPoint tx Co. Washington District of Columbia USA; ^4^ Department of Biomedical Engineering Johns Hopkins University School of Medicine Baltimore Maryland USA; ^5^ Johns Hopkins Translational Immuno Engineering Center, Translational Tissue Engineering Center, and Institute for Nanobiotechnology Johns Hopkins University Baltimore Maryland USA; ^6^ Departments of Neurosurgery, Oncology, Ophthalmology, Materials Science & Engineering, and Chemical & Biomolecular Engineering Johns Hopkins University School of Medicine Baltimore Maryland USA

**Keywords:** GBM, MSC, nanoengineering, nanoparticles, resection, stem cells

## Abstract

Confounding results of engineered mesenchymal stem cells (MSCs) used as cellular vehicles has plagued technologies whereby success or failure of novel approaches may be dismissed or inaccurately ascribed solely to the biotechnology platform rather than suitability of the human donor. Polymeric materials were screened for non‐viral engineering of MSCs from multiple human donors to deliver bone morphogenic protein‐4 (BMP4), a protein previously investigated in clinical trials for glioblastoma (GBM) to combat a subpopulation of highly invasive and tumorigenic clones. A “smart technology” that target the migratory and stem‐like nature of GBM will require: (1) a cellular vehicle (MSC) which can scavenge and target residual cells left behind after surgical debulking and deliver; (2) anti‐glioma cargo (BMP4). Multiple MSC donors are safely engineered, though varied in susceptibility to accept BMP4 due to intrinsic characteristics revealed by their molecular signatures. Efficiency is compared via secretion, downstream signaling, differentiation, and anti‐proliferative properties across all donors. In a clinically relevant resection and recurrence model of patient‐derived human GBM, we demonstrate that nanoengineered MSCs are not “donor agnostic” and efficacy is influenced by the inherent suitability of the MSC to the cargo. Therefore, donor profiles hold greater influence in determining downstream outcomes than the technical capabilities of the engineering technology.

**Translational Impact Statement**

This research underscores the pivotal role of donor‐specific traits in engineered mesenchymal stromal/stem cell (MSC) performance for glioblastoma (GBM) therapy. Evaluating multiple human MSC donors in delivering anti‐GBM cargo reveals varied susceptibility to accept the therapeutic payload due to the unique intrinsic characteristics of the donor. Emphasizing the impact of donor profiles over technical engineering capabilities, this study advocates for personalized approaches in treatments utilizing cellular therapies. Understanding donor‐specific influences on MSC efficacy highlights the necessity for tailored strategies to optimize therapeutic outcomes and enhance GBM management.

## 1 INTRODUCTION

Engineered mesenchymal stromal/stem cells (MSCs) are promising cell‐based therapy platforms for many diseases across regenerative medicine, neurodegeneration, and oncology.^1^, ^2^, ^3^ However, studies exploring the therapeutic potential of engineered MSCs have largely failed to account for donor heterogeneity.^3^, ^4^, ^5^ Most pre‐clinical studies use a single cell line or donor and extrapolate biotherapeutic outcomes to all MSCs.^6^, ^7^ Based on the International Society for Cell and Gene Therapy (ISCT) recommendation, MSC identity is satisfied when surface epitopes, multipotent potential, and plastic adherence is met.^8^ It is now understood that MSC donor variability is far greater than the minimum defined criteria holding larger implications on the success of cellular engineering technologies.^9^, ^10^, ^11^, ^12^ The authorized use of MSCs meeting minimum criteria has contributed to the conflicting and anecdotal evidence of MSC therapeutics and the irreproducibility of engineering strategies across the literature.^2^, ^13^, ^14^, ^15^, ^16^ More importantly, confounding results of MSC biology has plagued engineering technologies in regenerative medicine and oncological applications whereby the success or failure of novel experimental platforms may be dismissed or inaccurately ascribed solely to the engineering modality rather than the inherent biological differences among donors.^17^ There has been a recent surge in work suggesting that MSC heterogeneity, tissue source and manufacturing protocols play a far greater role than the technical capability of the biotechnology platform.^18^, ^19^ Studies have suggested that tissue source and manufacturing protocols is the biggest determining factor impacting MSC biology.^19^, ^20^ Given the demonstrated safety of MSCs as cell‐based delivery vehicles, the proper selection of optimal donors, prior to engineering, is a necessary step to advance the future of cell‐based therapy and protect transformative or innovative platforms with demonstrated potential.

This study assesses the utility of a universal engineering platform for cellular applications to advance the effectiveness of “off‐the‐shelf” engineering strategies for autologous applications. The development of a nonviral NP technology with universal applicability for “off‐the‐shelf” therapy can minimize variability of technical processes and reduce the introduction of significant changes during clinical biomanufacturing. Universal formulations can offer promise if they can accommodate the complexity of the intrinsic biological systems inherent to the donor cell source. When considering MSC nanoengineering strategies, it is unknown whether inherent molecular or functional drivers intrinsic to the MSC donors can be predictive of response prior to engineering and application. Similarly, it remains to be explored whether MSC donor heterogeneity holds greater influence in determining downstream outcomes than the technical capabilities of the cellular engineering platform and the therapeutic cargo it intends to deliver. This is important for *allogenic—*but especially important for *autologous* cell‐based therapies. Therefore, we investigate if inherent biological determinants of the MSC donor impact its susceptibility to accept a therapeutic cargo, such as bone morphogenic protein‐4 (BMP4), following non‐viral nanoparticle‐mediated genetic engineering using our poly (beta‐amino ester) (PBAE)‐based nanoparticle (NP) platform technology as previously described.^21^ We have demonstrated the safety of BMP4 by MSC delivery, showing that NPs outperforms lentiviral engineering and lipofectamine of the same MSC lines.^21^, ^22^ However, it is unknown to what extent the source of the MSC donor can influence therapeutic outcomes. To answer this, adipose‐derived MSCs from multiple human donors were non‐virally engineered to express BMP4 for the treatment of the most devastating human brain cancer, glioblastoma (GBM) (Study schematic).

**Figure:**
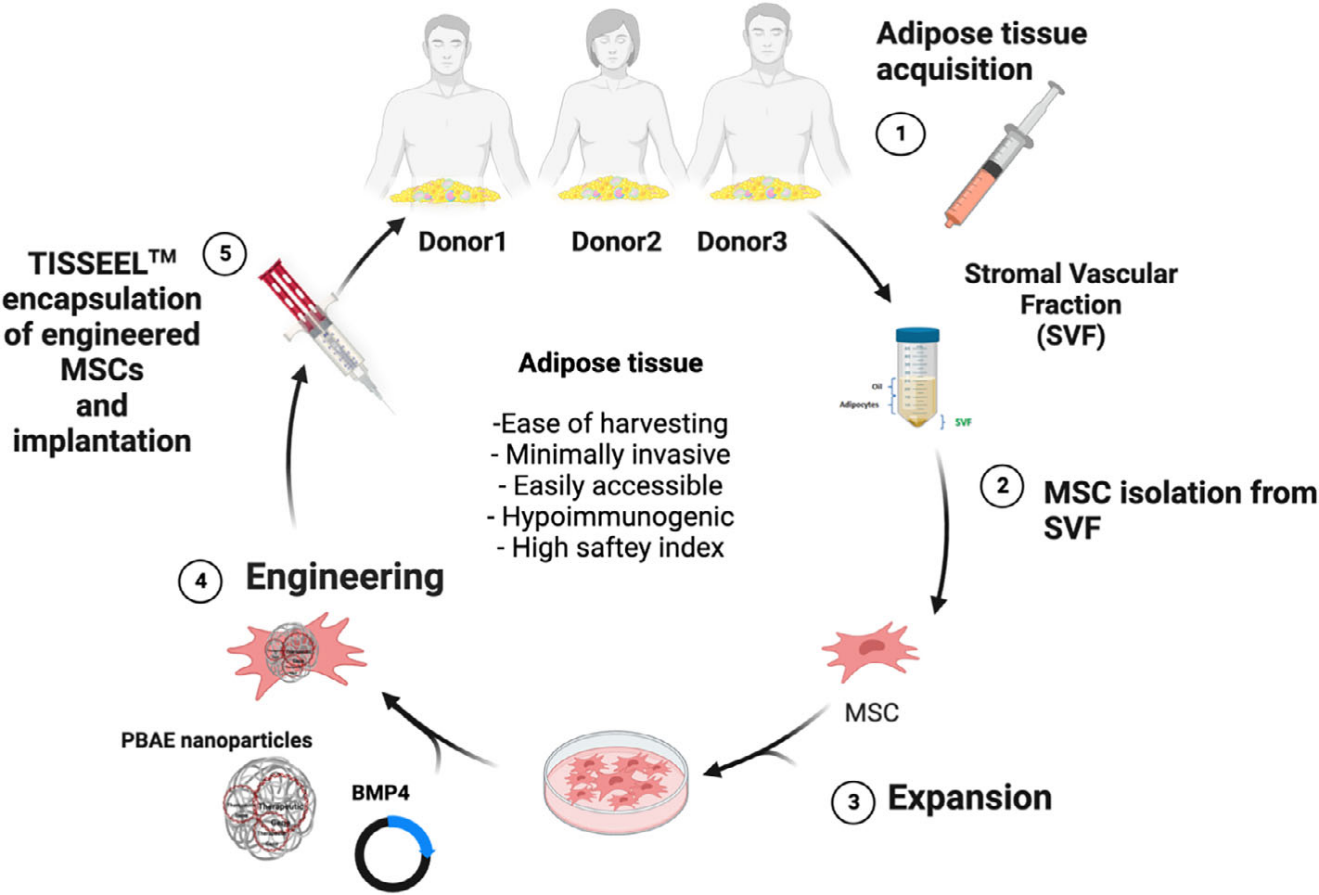
STUDY SCHEMATIC: Nanoengineering of patient‐derived stem cells. PBAE‐MSC engineering. SVF: stromal vascular fraction; MSC: Mesenchymal stem cells; BMP4: bone morphogenic protein 4; PBAE: Poly (beta‐amino ester).

GBM is the most aggressive and malignant type of primary brain cancer accounting for 50%–60% of all primary brain tumors with a 3.3%–10% five‐year survival rate.^23^, ^24^, ^25^ Despite the most aggressive standards of care (chemotherapy and radiation), residual brain tumor‐initiating cells (BTICs), displaying stem‐like properties, are often left behind after debulking and migrate away from the site of origin to repopulate, leading to the universal recurrence rate afflicting GBM patients today.^26^, ^27^ Thus, there is an urgent need to develop therapies that target highly migratory and invasive cancer cells in GBM, namely BTICs. BMP4 reduces the stem‐like properties of BTICs by driving them toward a committed glial lineage, thereby reducing their capacity to self‐renew and proliferate.^28^, ^29^ A large study of patients (*n* = 630) revealed that BMP4 expression was significantly lower in gliomas than healthy patient tissue.^30^ The same study also demonstrated that patients with high BMP4 expression had substantially better prognosis. Multiple studies have demonstrated that recombinant human BMP4 (rhBMP4) holds potent therapeutic impact for GBM and was investigated in clinical trials for recurrent disease (NCT02869243).^28^, ^31^, ^32^ Similarly, MSC‐encapsulated in fibrin glue (TISSEEL),^33^ a gel widely used in neurosurgery to achieve hemostasis, is currently being explored in a phase I non‐randomized clinical trial for recurrent GBM post‐surgical debulking.^34^ For this reason, we tested the combination of both applications to advance the future of cellular biotechnology by using a clinically relevant model, for the first time, to mimic the human clinical paradigm of recurrent GBM.

TISSEEL encapsulated BMP4‐engineered MSCs from multiple human donors are evaluated for their suitability to deliver BMP4 through non‐viral engineering. To our knowledge, this is the first study to explore the influence of donor intrinsic cues on capability to secrete a therapeutic cargo, such as BMP4, via non‐viral engineering. We assessed outcomes of tropic and migrative capacity to BTICs, anti‐proliferative and anti‐glioma effects on BTICs, and downstream effector signaling across multiple engineered donors to elucidate the impact of donor characteristics. Upon associating MSC donor properties to engineering potential enabled by gene expression arrays, we evaluated the clinical utility of this platform on survival outcomes.

We surmise herein that the technical success of genetic modification and therapeutic outcomes across donor MSCs is necessary, but not sufficient, to ensure high potency as a cellular therapeutic for GBM. We posit that donor characteristics plays an independent role on efficacy, beyond the influence of the engineering platform. Identifying optimal donor parameters is critical for the future of bioengineering cellular strategies, especially for orphan diseases, such as GBM, where the selection of donors is critical, costly, and time dependent.

## 2 RESULTS

### 2.1 Robust NP‐based reprogramming depends on donor intrinsic MSC characteristics

**FIGURE 1:**
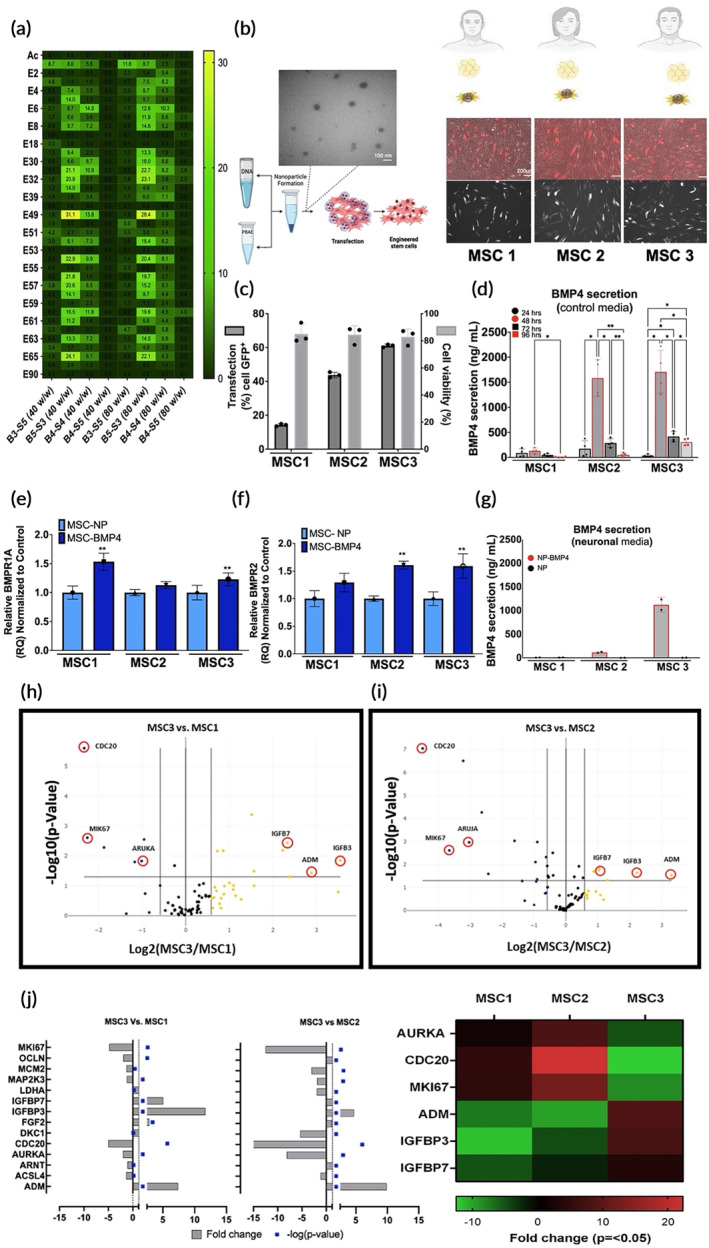
Robust NP‐based reprogramming depends on donor intrinsic MSC characteristics. (a) PBAE library screening of donor lines for optimal transfection. (b) Schematic of MSC isolation from donor and NP preparation. Representative transmission electron microscopy (TEM) and fluorescence microscopy images (mCherry) of MSC transfected with top NP formulation “4‐4‐6” (scale bar is 100 nm for TEM and 200 μm for fluorescence images). (c) Transfection efficiency and viability of MSCs engineered with “4‐4‐6”. (d) Concentration of human BMP4 secreted over a 96‐h time‐course post MSC engineering in MSC medium. (e) Quantitative PCR showing mRNA levels of BMPR1A receptor on donor MSCs post‐engineering. (f) Quantitative PCR showing mRNA levels of BMPR2 receptor on donor MSCs post‐engineering. (g) Concentration of BMP4 secreted at 48 h after MSC engineering in neuronal medium. (h) Volcano plot of MSC3 as treated group and MSC1 as control; red circles indicate overexpressed genes found in MSC1 by plotting log2 of fold change on *x*‐axis against their statistical significance on *y*‐axis. (i) Volcano plot of MSC3 as treated group and MSC2 as control; red circles indicate overexpressed genes found in MSC2 by plotting log2 of fold change on *x*‐axis against their statistical significance on *y*‐axis. (j) Plot and heat‐map of significantly regulated genes in MSC3 compared to MSC1 and MSC2 (−log(*p*‐value) > 1.2; fold change = *p* < 0.05). Data represent mean ± standard deviation (SD) of three independent experiments pooled and replicated at least twice or more. Statistics by analysis of variance (ANOVA) with Tukey's multiple comparison post‐hoc analysis. *p < 0.05, **p < 0.01, ***p < 0.001, compared to the transfection control (NP). ADM, adrenomedullin; AURKA, aurora kinase A; CDC20, cell division cycle 20; IGFB, insulin like growth factor binding protein; MKI67, marker of proliferation Ki67. Scale in (b) is 100 nm TEM and (b) 200 μm for fluorescence.

To determine the impact of donor heterogeneity on the engineering platform, three primary human derived MSC donors (MSC1, MSC2, and MSC3) were screened against a library of PBAE NP formulations (Table S1). Green fluorescent protein (GFP) was used for polymer screening and lyophilized particles with mCherry was used for donor screening (Figure 1a). NP formulation “4‐4‐6” consisting of the 4‐4‐6 PBAE (Figure S1) complexed with plasmid DNA, was selected for further study as it satisfied the following criteria: (i) transfection efficiencies from the same formulation across multiple donors; (ii) low toxicity upon uptake; (iii) high viability; and (iv) no significant modification or customization for each individual donor (broad applicability for autologous engineering) (Figure 1b,c). To assess the innate impact of donor characteristics on cellular engineering, MSCs were engineered with 4‐4‐6 NPs expressing mCherry or BMP4 under the same vector backbone and promoter (Figure S2). Conditioned medium was collected at different time‐points, and BMP4 was quantified. In accordance, MSC1 secreted the lowest concentration of BMP4 (~134 ng/mL), while MSC2 and MSC3 secreted much higher levels (1265 and 1360 ng/mL, respectively) (Figure 1d). BMP4 secretion peaked at 48 h and diminished at 96 h post‐transfection across all donors, demonstrating consistent secretion timeframe (Figure 1d).

MSCs respond to environmental cues via paracrine and autocrine signaling.^35^ Therefore, BMP4 receptor expression at peak time‐point (48 h) was investigated. BMP4 binds to bone morphogenetic protein receptor (BMPR)1A and engages with BMPR2 to preferentially activate canonical downstream signaling via SMAD.^36^ Compared to the transfection control (MSC‐NP), MSC1 transfected with BMP4 (MSC‐BMP4) saw an increase in BMPR1A transcript levels (Figure 1e), while BMPR2 remained unchanged (Figure 1f). On the contrary, MSC2‐BMP4 displayed a substantial increase in BMPR2 expression, while BMPR1A levels in MSC2 remained unchanged (Figure 1f,g). In the case of MSC3‐BMP4, both BMPR1A and BMPR2 were increased compared to the transfection control (Figure 1f,g). While MSC2 and MSC3 displayed similar levels of BMP4 secretion (Figure 1d), only MSC3 displayed a concomitant increase in both BMPR receptors upon engineering, demonstrating that donor response to the secreted cargo is differentially influenced by the donors.

To test the utility of this platform technology in an environment mimicking GBM, BMP4 secretion levels were evaluated in the presence of neuronal supplements (Gem21 neuroplex medium; primarily containing insulin, transferrin, and progesterone)^37^ at 48 h. MSC1 displayed over a 20‐fold reduction in BMP4 secretion, measuring at 6.1 ng/mL, which was equivalent to the transfection control **(**Figure 1g). Similarly, MSC2 displayed a 10‐fold reduction in BMP4 secretion upon exposure to neuronal supplements, however levels were still substantially higher (115 ng/mL) than MSC2‐NP controls. MSC3‐BMP4 was the only donor that maintained BMP4 levels above 1000 ng/mL (1121 ng/mL) in both MSC and neuronal medium, furthering the findings that MSC donor heterogeneity and environmental cues substantially impact potency beyond the NP or engineering approach (Figure 1d,g). In the case of BMP4‐engineered MSC1 and MSC2, secretion profiles were drastically reduced upon exposure to the neurobasal medium, suggesting that innate factors of the MSC can oppose the success of cellular engineering strategies, holding large implications on the precision of potency assays for clinical translation. Notably, heterogeneity in terms of susceptibility to a reduction in transgene expression induced through environmental changes was observed among the three donors. Incongruency in the efficacy of MSC‐engineering platforms across laboratories may be due to variation in donors, the medium in which cells are in, and the susceptibility of the donor to the transfection and secretion of the specific cargo itself (BMP4). Outcomes may explain the variances in patient‐response or therapeutic outcomes across studies investigating MSC application.

Notably, MSC3 maintained BMP4 secretion despite changes in medium. A pathway gene‐expression array was performed to explore the inherent differences in MSC1 (Figure 1h) and MSC2 (Figure 1i) relative to MSC3, which maintained BMP4 levels despite changes in medium. Results indicate that genes related to insulin signaling are differentially expressed in MSC3 versus other donors. Insulin is an important component of neurobasal medium^37^ and insulin signaling is upregulated in GBM promoting survival and proliferation of malignant cells.^38^ Not surprisingly, MSC3 showed enrichment of insulin like growth factor binding protein (IGFB)7 compared to MSC1 and MSC2 (Figure 1j). IGFB7 is a known inhibitor of insulin signaling with the highest binding affinity to insulin among all members of the IGFBP family.^39^, ^40^ Additionally, IGFBP3 and adrenomedullin (ADM), which have inhibitory effects on the insulin signaling pathway^41^, ^42^, ^43^ were differentially upregulated in MSC3. Insulin activates the mammalian target of rapamycin (mTOR),^44^, ^45^ which has previously been linked to the suppression of BMP signaling,^45^ indicating that high expression of IGFBP7, IGFB3, and ADM in MSC3 may contribute to the preservation of BMP4 secretion in insulin‐rich medium (Figure 1d,g). MSC3 also displayed decreased expression of aurora kinase A (AURKA), cell division cycle 20 (CDC20), and marker of proliferation Ki‐67 (MKI67) compared to MSC1 and MSC2 (Figure 1j) suggesting that energy outputs may be expended on cell division, rather than exogenous gene expression imposed by the engineering platform. Taken together, BMP secretion depends on the innate regulatory mechanisms of the donor and the environmental cues. Further studies are necessary to identify the most valuable predictors of BMP4 secretion compatibility in MSC donors.

This finding extends beyond BMP4 cargo and affects exogenous gene transfer to MSCs more generally. Strategies to screen for pathways that interfere with MSC cellular engineering approaches are likely to improve therapeutic outcomes in engineering platforms. Our group has previously shown that NP‐mediated delivery of BMP4 outperforms viral‐engineering and lipofectamine of the same MSC line.^21^, ^22^ To our knowledge, this is the first finding to indicate that intrinsic donor profiles and medium in which they are in may hold greater influence in determining downstream outcomes than the technical capabilities of the PBAE engineering technology.

### 2.2 BMP4 engineering enhances the migratory potential of MSCs

**FIGURE 2:**
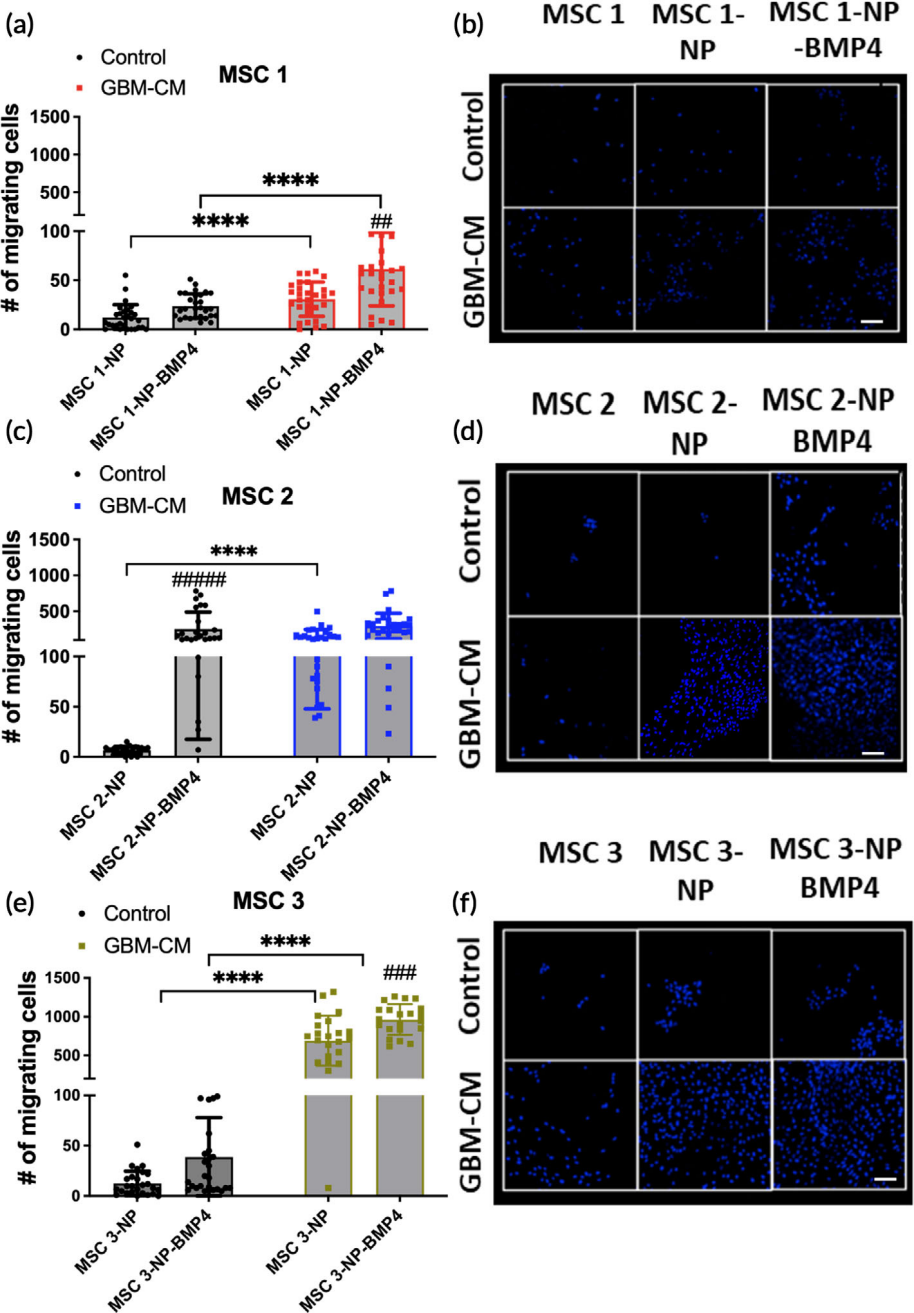
BMP4 engineering enhances the migratory potential of MSCs. Engineered MSCs were seeded on the top chamber of transwells, and the bottom chamber was filled with GBM‐conditioned medium or MSC medium supplemented with 5% FBS to induce migration. (a) Migration analysis of MSC1 toward control and GBM‐conditioned media over 24 h. (b) Confocal images representative of MSC1 migration toward control and GBM‐conditioned medium quantified by DAPI. (c) Migration analysis of MSC2 toward control and GBM‐conditioned media over 24 h. (d) Confocal images representative of MSC2 migration toward control and GBM‐conditioned medium quantified by DAPI. (e) Migration analysis of MSC3 toward control and GBM‐conditioned media over 24 h. (f) Confocal images representative of MSC3 migration toward control and GBM‐conditioned medium quantified by DAPI. Data represent mean ± standard deviation (SD) of three independent experiments pooled and replicated at least twice or more. Statistics by ANOVA with Tukey's multiple comparison post‐hoc analysis. *p < 0.05, ***p < 0.001, ****p < 0.0001, ##*p* < 0.01, ###*p* < 0.001, and ####*p* < 0.0001 compared to the transfection control (NP) under the same conditions. BTICs, brain tumor‐initiating cells; CM, conditioned medium; GBM, glioblastoma; control, MSC medium; ns, not significant; SDF‐1, stromal cell‐derived factor 1. Scale in (e), (g), and (i) represent 100 μm taken at 10×.

Given the large differences in secretion profiles among the donors changes pertinent to MSC applications and cargo delivery, such as migration, were evaluated post‐engineering. Previously, it was determined that approximately 100 ng/mL of BMP4 is sufficient to induce an anti‐glioma response against BTICs.^28^ Therefore, the low‐secreting (MSC1; ~6 ng/mL) and medium‐secreting (MSC2; 115 ng/mL) cell lines were compared by assessing stromal cell‐derived factor‐1 (SDF‐1), a major component of the MSC migration axis to GBM.^46^, ^47^ SDF‐1 was found to be significantly over‐expressed and secreted in MSC2‐BMP4 compared to the transfection control, while this increase was not observed in the low‐BMP4 secreting MSC1‐BMP4 line (Figure S3a–c). To determine if the increase in SDF‐1 was correlated with improved migratory potential, a migration assay using transwell containing MSC medium or GBM conditioned medium (GBM‐CM) was performed. In control medium, BMP4‐engineering failed to enhance the migrative potential of MSC1 (Figure 2a,b), while MSC2‐BMP4 displayed substantially increased migration compared to the transfection control (MSC2‐NP) (Figure 2c,d), consistent with SDF‐1 levels (Figure S3b). Among all three lines, MSC3 showed the highest increase in migration when the assay was performed with GBM‐conditioned medium **(**Figure 2e,f). Overall, BMP4‐engineering enhanced the migratory potential of all MSCs donors toward GBM‐medium.

To our knowledge, this is the first study to demonstrate that, in addition to serving as an anti‐glioma cargo, BMP4 can boost the innate migratory capacity of MSCs which could be utilized to enhance the migration for other applications requiring timely delivery (Figure S3a–c). However, heterogeneity in terms of BMP4‐induced migration was observed among the three cell lines demonstrating that each engineered‐MSC possesses its own migratory capabilities that may be dependent on secretion profiles and should be considered prior to therapeutic implementation. Nonetheless, BMP4 engineering of MSCs is shown to enhance the migration of all donors toward GBM‐conditioned medium.

### 2.3 MSC‐BMP4 override the mitogenic effects of EGF and FGF but differentially induces BTIC lineage commitment

Previous studies have demonstrated that rhBMP4 reduces the proliferative capacity of BTICs and their tumor forming abilities in vivo by diminishing the frequency of asymmetric cell division in cluster of differentiation (CD)133‐positive glioma‐initiating cells.^28^ Similarly, genetically modified MSCs engineered to express BMP4 have demonstrated survival benefits in animal models of GBM.^21^, ^22^ Furthermore, some studies have demonstrated that anti‐glioma effects were observed with unmodified MSCs,^22^ albeit to a lesser extent, suggesting that MSCs have anti‐glioma benefits beyond their ability to deliver the therapeutic cargo. To our knowledge, this is the first study to evaluate the impact of MSC‐BMP4 donor heterogeneity on BTIC self‐renewal and differentiation capacity.

**FIGURE 3:**
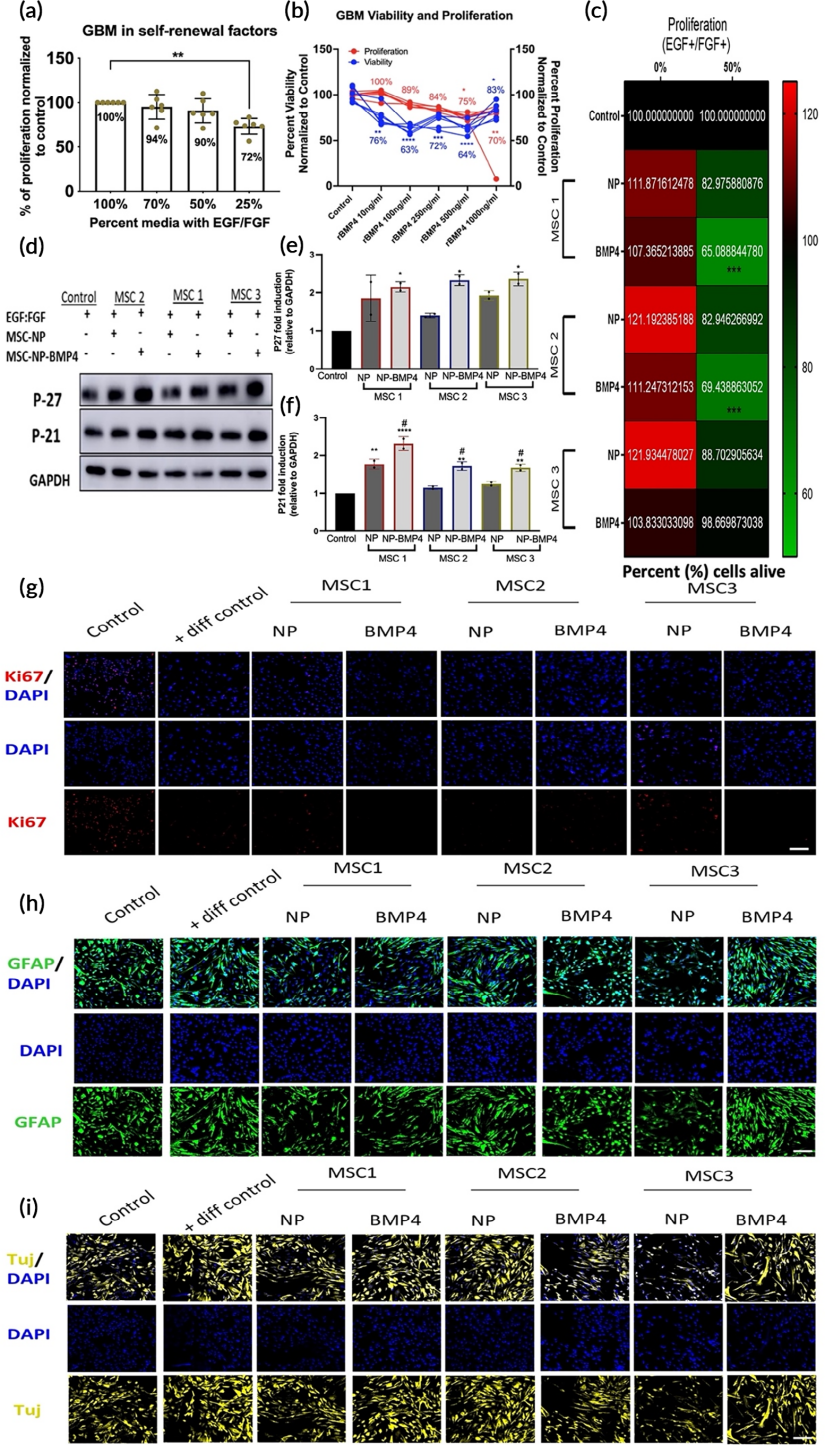
MSC‐BMP4 override the mitogenic effects of EGF and FGF but differentially induces BTIC lineage commitment. (a) BTIC viability through MTT assay was measured by supplementing medium with increasing amounts of EGF/GFG mitogens up to 100% supplementation (0.20 and 0.10 ng/mL) for 96‐h to identify critical concentration at which viability is not decreased to maintain the stem‐like phenotype. (b) BTIC viability (MTT) and proliferation (CyQuant) was measured with increasing supplementation of rhBMP4 for 96‐h. (c) Live BTIC count (CyQuant) upon MSC treatment was measured in the presence of EGF/FGF at 100% standard concentration or 50% concentration to determine impact of treatment on the stem‐like phenotype. (d) Representatives immunoblot of P27 and P21 protein expression evaluated in BTICs treated with MSC donors in the presence of 50% mitogen supplementation after 96‐h. (e) Quantitative measurement of P27 immunoblot expression in BTICs upon MSC treatment. (f) Quantitative measurement of P21 immunoblot. (g) Representative images of BTICs cultured in control (50% mitogens), differentiation control (10% FBS) or MSC1 donor treatments (1:1 dilution of media) for 10–14 days and stained with Ki67, (h) GFAP and (i) TUJ co‐localized with DAPI. Statistics by ANOVA with Tukey's multiple comparison post‐hoc analysis. *p < 0.05, **p < 0.01, ***p < 0.001, and ****p < 0.0001 compared to the control unless otherwise indicated. #*p* < 0.05 compared to the transfection control (NP). Data represent mean ± SD of three independent experiments pooled and replicated at least twice or more. Statistics by ANOVA with Tukey's multiple comparison post‐hoc analysis. BTIC, brain tumor initiating cell; diff, differentiation; EGF, epidermal growth factor; FGF, fibroblast growth factor; rh, recombinant human. Scale bars in (g–i) 100 μm.

To ensure the BTIC subpopulation was selected and maintained throughout the study, the addition of mitogens was sustained by continued supplementation of epidermal growth factor (EGF) for increased proliferation, and fibroblast growth factor (FGF) for enhanced self‐renewal.^48^ With 50% of the medium containing standard levels of mitogens, BTICs retain 90% of their proliferative capacity (Figure 3a), therefore, subsequent assays were performed under 50% conditions to maintain BTIC self‐renewal and proliferation. Prior to assessing the impact of MSC‐secreted BMP4 on the viability and proliferation of a patient‐derived BTIC line, the effects of rhBMP4 were determined. Exposure to rhBMP4 at doses less than 500 ng/mL did not significantly change the proliferation capacity of BTICs, while all doses (10–1000 ng/mL) reduced cell viability (Figure 3b). Notably, a 1000 ng/mL dose of rhBMP4 increased the viability from 64% to 83% at 500 ng/mL, though still significantly lower than controls (Figure 3b), highlighting the importance of concentration‐dependent effects previously observed.^18^, ^49^ To evaluate MSC‐donor heterogeneity on BTIC survival and if engineering compounded outcomes, a live‐dead assay was performed with or without the presence of mitogens. Bona‐fide BTICs retaining a stem‐like phenotype was evaluated against BTICs after mitogen withdrawal, representing the non‐stem population of GBM. Under the presence of mitogens, the secretome from the MSC transfection controls (MSC‐NP) did not cause a significant change in cell counts, while BMP4‐engineered MSC1 and MSC2 resulted in significant reduction (Figure 3c). Notably, MSC3, the highest BMP4‐secreting donor (>1000 ng/mL; Figure 1d,g) did not result in significant changes (Figure 3c), which mirrored the effects of rhBMP4 protein at the same dose (Figure 3b). All MSC‐BMP4 donors failed to produce a significant impact of the non‐stem‐like population (selected by the removal of mitogens) (Figure 3c), confirming the preferential effects of the platform on the stem‐like population of GBM. Notably, recent findings have demonstrated that BMP4 can impart conflicting effects in driving GBM malignancy,^30^ either promoting glioma growth by enhancing proliferative capacity^50^ or inducing quiescence.^51^ The dual role of BMP4 in self‐renewal and differentiation is likely due to concentration‐dependent effects. Therefore, the impact of donor heterogeneity on the propensity to secrete BMP4 may affect therapeutic activity and should be considered for future BMP4 dosing regimens.

The surviving and remaining BTICs were further assessed for cell cycle progression via p27 and p21 cell‐cycle inhibitors of cyclin‐dependent kinases (CDK). Low levels of p27 in GBM are associated with a worse prognosis and increased proliferation.^52^, ^53^, ^54^, ^55^ All donor MSCs engineered with BMP4 caused a statistically significant increase in p27 and p21 expression (Figure 3d–f). None of the donor cell lines (transfection controls) alone resulted in significant differences in p27 and p21 expression, except for MSC1, which increased p21 (Figure 3d–f). Notably, MSC1‐BMP4 induced the highest change of p21 in BTICs compared to untreated cells (Figure 3d–f), despite being the lowest BMP4‐secreting cell line. Data suggests that the endogenous impact of the donor can be more predictive of outcomes than the capability of the engineering platform and the cargo delivered.

The enhanced expression of p27 and p21 suggests cell‐cycle inhibition. To evaluate if the increases in cyclin‐dependent inhibitors resulted in post‐mitotic arrest, BTICs were conditioned with MSC medium for 10–14 days; results demonstrate potent suppression of proliferation via Ki67 (Figure 3g), consistent with the observed suppression cell‐cycle arrest (Figure 3c–f). Furthermore, a decrease in proliferation and increase of CDK inhibitors is likely to be associated with lineage commitment. It is known that MSCs differentiate neural stem cells (NSCs) and BTICs into neuronal or astroglial fates,^56^ and BMP4 supplementation is expected to enhance the latter. BMP4‐engineered donor cells displayed variant propensities to differentiate BTICs to astroglia, as evidenced by enhanced glial fibrillary acidic protein (GFAP) expression and morphological changes. In the case of MSC1‐BMP4, differentiation resembled its control counterpart (MSC1‐NP) (Figure 3h). On the contrary, BMP4 engineering of MSC2 and MSC3 resulted in increased astroglial commitment compared to the transfection control (Figure 3h). This is not surprising as both MSC2 and MSC3 secrete >100 ng of BMP4. Commitment to neuronal lineage was evaluated in parallel via neuron specific class III tubulin (TUJ). Neuronal differentiation of BTICs was observed with MSC1‐BMP4 (Figure 3i), suggesting that low secretion of BMP4 may not have been sufficient to induce the preferential commitments to the astrocytic fate. On the contrary, MSC2‐BMP4 displayed a substantial decrease in TUJ1 expression compared to the transfection controls furthering the observed preferential GFAP commitment (Figure 3h,i).

### 2.4 Downstream BTIC signaling is the result of the combined influence of the cargo and MSC donor‐intrinsic mechanisms

BMP4 reduces the stem‐like population of GBM by inducing cell‐cycle arrest or lineage commitment through the engagements of differential receptor dimerization.^57^, ^58^ Understanding of BMP receptors come from studies on NSCs, as BTICs display NSC properties.^59^ Lineage tracing and trajectory analysis have revealed a close stem‐like network in gliomas believed to be the event of a multi‐step transcriptional reprogramming of NSC‐like cells, marking the neurogenic to glioma‐genic switch as an early precursor to brain oncogenesis.^60^ BMP receptors regulate downstream gene expressions in NSCs, and the relative contribution of the BMP cognate receptors is dependent on the influence of two type I receptors, BMPR1A (ALK3) and BMPRIB (ALK6). The receptors form a tetrameric complex with type II receptor (BMPR2),^36^ and downstream signaling is dependent on the combination of ligand–receptor pair signaling that is likely to aid in the development of screening tools for donor selection of BMP4 delivery.

**FIGURE 4:**
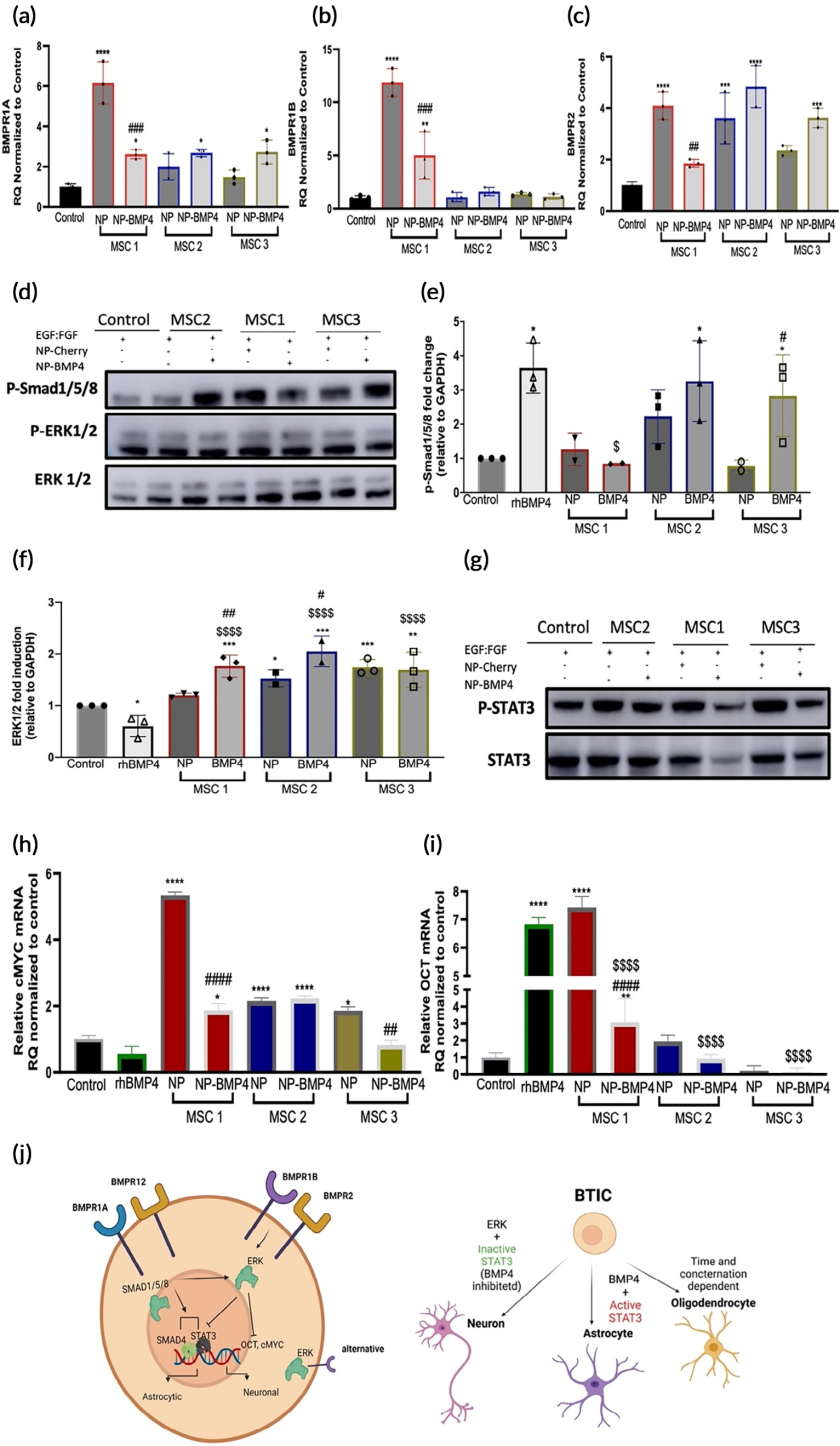
Downstream BTIC signaling is the result of the combined effect of the cargo and MSC donor mRNA expression of (a) BMPR1A, (b) BMPR1B and (c) BMPR2 in BTICs after 96‐h treatment of MSC donor conditioned medium. (d) Representatives immunoblot of pSMAD1/5/8, ERK in BTICs after 96‐h of donor MSC treatments. (e) Quantitative immunoblot measurements of pSMAD1/5/8 phosphorylation. (f) Quantitative immunoblot measurements of ERK phosphorylation in BTICs. (g) Representatives immunoblot of STAT3 in BTICs after 96‐h of donor MSC treatment. (h) mRNA expression of cMYC in BTICs after 96‐h treatment of MSC donor conditioned medium. (i) mRNA expression of OCT in BTICs after 96‐h treatment of MSC donor conditioned medium. (j) Schematic of proposed mechanisms. Data represent mean ± SD of three independent experiments pooled and replicated at least twice or more. Statistics by ANOVA with Tukey's multiple comparison post‐hoc analysis; blot comparisons were corrected for multiple comparisons by controlling for false discovery rate (FDR), *p < 0.05, **p < 0.01, ***p < 0.001, and ****p < 0.0001 compared to the control. #*p* < 0.05, ##*p* < 0.01, ###*p* < 0.001, and ####*p* < 0.0001 compared to the transfection control (NP). $*p* < 0.05, $$*p* < 0.01, $$$*p* < 0.001, and $$$$*p* < 0.0001 compared to recombinant human BMP4 (rhBMP4).

We evaluated the extent of engineered MSC donor variability on the engagement of specific BMP4 receptor pairings in BTICs. While the conditioned medium of all three BMP4‐engineered MSC donors caused a significant increase in BMPR1A in BTIC compared to untreated control (Figure 4a), only engineered MSC1 concomitantly increased BMPR1B receptor expression (Figure 4b). Additionally, BMP4‐engineered MSC2 and MSC3 caused a significant upregulation of BMPR2 in BTICs, indicative of downstream activation, while MSC1‐BMP4 did not (Figure 4c). In the case of the transfection controls, a substantial increase in BMPR1A and BMPR1B was imparted by MSC1‐NP, while BMPR2 was increased by MSC2‐NP; receptors remained unchanged in response to MSC3‐NP (Figure 4a–c). BMPR1B has previously been shown to result in neural commitment of NSC.^29^, ^61^ The increase in BMPR1B in MSC1‐BMP4 is consistent with the differentiation patterns observed in BTICs upon treatment (Figure 3h,i). BTICs treated with MSC1‐BMP4 opposed BMP4 activation of BMPR1A and BMPR2 (Figure 4a–c) suggesting that the BMP4 engineering approaches may activate antagonistic cues, potentially due to the donor cells, which may oppose canonical downstream regulators involved in GFAP commitment, as seen during differentiation (Figure 3h,i). Therefore, it is important to note that the endogenous secretome of MSCs may, in certain cases, cause significant changes in pathways that are targeted through engineering strategies, potentially resulting in variable downstream cues related to BMP4 signaling in BTICs.

To understand the impact of downstream activation imparted by the donor line (beyond the BMP4 cargo), we analyzed downstream effector signaling upon BMPR engagement; both the canonical and non‐canonical BMP4 pathways were assessed. Preferential engagement of BMPR1A/BMPR2 activates SMAD1/5/8 by serine‐phosphorylation‐related downstream events while BMPR1B/BMPR2 regulates cellular processes through SMAD‐independent pathways mediated through MAPK/ERK activation (Figure 4d), consistent with previously observed signaling related to BMP4.^62^ Specific pathways activated in response to therapy are likely dependent on both BMP4 and other extracellular cues inherently imposed by the MSC donor.

Considering the differential engagement of the BMP receptors, BTIC downstream signaling of SMAD1/5/8 and ERK1/2 were evaluated. BMP4‐engineered MSC‐2 and MSC‐3 activated SMAD1/5/8, mirroring the effects of rhBMP4 (Figures 4e and S4 **)** and secretion profiles (Figure 1d,g). Consistent with an increase in BMPR1B expression, MSC1‐BMP4 exclusively activated ERK1/2 (Figure 4f). Notably, all three BMP4‐engineered MSC donor secretome activated ERK1/2, while rhBMP4 alone failed to do so (Figure 4d and S5), suggesting donor intrinsic activation programs, independent of cargo are at play. Similarly, activation of the non‐canonical pathway was also seen in the transfection controls, implicating a BMP4‐independent mechanism (Figure 4d,f). STAT3 was assessed to further understand the effects of downstream signaling. STAT3 is implicated in GBM aggressiveness as well as BTIC differentiation. All transfection controls enhanced STAT3 while BMP4 engineering reversed STAT3 overexpression (Figure 4g), providing an additional therapeutic benefit that maintains the delicate balance required to curb malignancy from donor‐imposed interference. Overall, the activation of SMAD reflected the uptake and secretion mechanisms of the cargo, while ERK1/2 activation is dependent on the donor; the combined effect of these activation programs influenced BTIC outcomes. Findings implicate the influence of the cellular vehicles above the engineering and cargo, suggesting that therapeutic and engineering methods must select for proper donors prior to engineering to ensure the reproducibility, precision, and success of such technologies.

The implication of BTIC self‐renewal upon receptor activation and downstream signaling was investigated through gene expression of known regulators involved in pluripotency and GBM malignancy. cMYC and octamer‐binding transcription factor 4 (OCT4) are involved in GBM self‐renewal, yet play pivotal roles in growth control, apoptosis, and differentiation.^63^ All transfection controls enhanced cMYC (Figure 4h), while BMP4 engineering of MSC1 and MSC3 reversed cMYC overexpression. On the contrary, MSC2‐BMP4 failed to reproduce these effects, further highlighting donor heterogeneity and the impact of intrinsic factors (Figure 4h). A similar effect was evident in MSC1‐BMP4 and MSC3‐BMP4 for OCT4 expression. Specifically, BTICs treated with rhBMP4 resulted in an overexpression of OCT4, while BMP4 engineered MSCs downregulated OCT4 (Figure 4i). Complex inhibitory cues can be imparted by the cellular engineering approach itself resulting in outcomes that oppose the expected consequence of the delivered cargo, BMP4. Our findings demonstrate that variable downstream signaling activates differentiation mechanisms and implicate STAT3 at the crux of GFAP commitment (Figure 4j). Further studies are needed to qualify which MSC factor overrides the therapeutic cargo upstream of STAT3.

### 2.5 Safety of MSC encapsulation in TISSEEL for local implantation in surgical cavity

**FIGURE 5:**
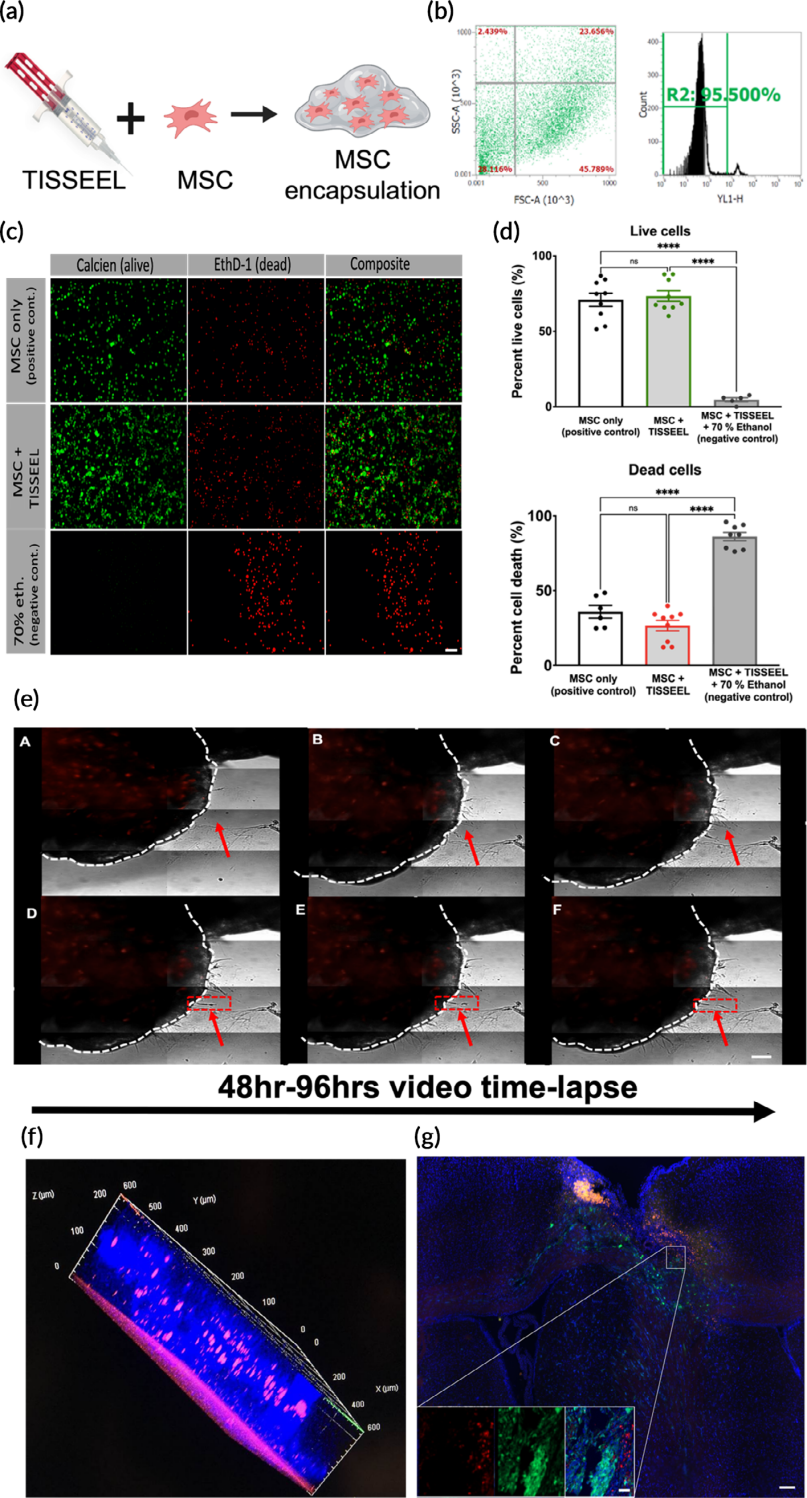
Safety of MSC encapsulation in TISSEEL for local implantation in surgical cavity. (a) Schematic representation of MSC and TISSEEL encapsulation and application paradigm. (b) Flow cytometric analysis measuring viability of MSCs encapsulated in the TISSEEL‐gel component (thrombin) prior to dilution using propidium iodide. (c) Representative confocal image of live/dead assay of MSCs loaded into the dual set syringe ejected out of tip. (d) Live staining (calcein green) and dead (ethidium homodimer 1‐red) were used to evaluate safety and toxicity of MSCs encapsulated in TISSEEL fibrin gel immediately after ejection. (e) MSCs‐NP (mCherry) were encapsulated in a TISSEEL bubble (white dashed line) and incubated in 2 mL medium. Cells were imaged via time‐lapse microscopy for 48–96 h. MSCs remain viable as demonstrated by red fluorescent surrogate marker (e:A) extend their processes; red arrows (e:B,C), migrate out of TISSEEL (e:D), divide (e:E—dashed rectangle) and continue to travel (d:F—dashed rectangle). Dashed white outlined—TISSEEL; red arrows—encapsulated MSCs extending their processes; red dashed rectangle—MSCs escaping TISSEEL (f) MSCs‐mCherry encapsulated in TISSEEL fibrin glue escape TISSEEL and penetrate live organotypic mouse brain slice (250 μm) imaged by confocal to localize MSCs‐mCherry/DAPI (pink). (g) Representative immunofluorescence tissue slice of MSCs encapsulated in TISSEEL and implanted in the resection cavity after tumor de‐bulking. Inset figures represent individual fluorescent channels demonstrating MSC migration marked with (mCherry) in proximity to GBM cells (GFP). Green: GBM; blue: nuclei stained by DAPI. Data represent mean ± SEM of at 2–3 pooled replicates or more. Statistics by ANOVA with Tukey's multiple comparison post‐hoc analysis. *p < 0.05, **p < 0.01, ***p < 0.001, and ****p < 0.0001. Scale in: (c) 200 μm, (e) 400 μm, (g) large image 800 μm, and inset 100 μm.

To further the impact of this study for clinical translation, engineered MSC cell‐based therapy was assessed for the local application of post‐surgical GBM. MSCs encapsulated in TISSEEL fibrin gel was done to maintain cells in the surgical cavity following current clinical approaches for MSC therapy (Figure 5a). The encapsulation study is intended to repeat the clinical paradigm of a phase I ongoing study on the use of MSCs for GBM.^34^ Cytotoxicity of MSCs in TISSEEL was tested in a diluted mixture of lactated ringer's solution (LRS) and thrombin to represent the components of the application prior to gelation and implantation following the clinical protocol; MSCs remained 95% viable (Figure 5b). Post‐gelation, MSCs (5–25 × 10^3^ cells/μL) were ejected out of the applicator and viability and cell death assays were performed. MSCs in TISSEEL remain alive and viable, demonstrating no significant change compared to non‐encapsulated MSCs (Figure 5c,d). To illustrate if MSCs retain their migratory potential and are able to escape out of TISSEEL, MSCs were live imaged and followed using time‐lapse microscopy for 48–96 h (Video S1), demonstrating that MSC‐NPs remain active (Figure 5e:A,B), extend their processes in preparation for escape (Figure 5e:C), escape TISSEEL encapsulation (Figure 5e:D), and continue to migrate and travel (Figure 5e:F), showing preserved MSC migratory capacity post‐encapsulation.

Furthermore, an organotypic live mouse brain slice model representing the 3D cytoarchitecture of the brain was used to investigate the capability of MSCs to escape encapsulation and penetrate a live brain slice. MSCs (0.25 × 10^6^) encapsulated in TISSEEL were shown to successfully infiltrate the live tissue after 72 h, demonstrating successful migration and tissue penetration (Figure 5f). To assess the utility of locally applied MSC‐encapsulated TISSEEL in recurrent model of GBM, MSCs labeled with mCherry were injected into the resection cavity post GBM debulking and animals were sacrificed. MSCs escaped TISSEEL encapsulation, and preferentially migrated along the spatial trajectory of BTICs, resulting in co‐localization with distant migratory cells (Figure 5g). Outcomes demonstrate the successful utility and migratory ability of engineered TISSEEL‐encapsulated MSCs toward BTICs in a clinically relevant resection and recurrent model, mimicking the human paradigm for locally applied cell‐based therapy.

### 2.6 Technical success of MSC1‐BMP4 in TISSEEL is necessary, but not sufficient, for therapeutic potency in a clinically relevant resection model of human GBM

To advance precision medicine of cellular applications, the process of identifying appropriate and therapeutically active MSC donors must account for cell intrinsic factors or unique parameters that would reflect their propensity to enhance the efficacy of the engineering platform and the chosen cargo. Furthermore, previous studies have demonstrated opposing results showing that MSCs suppress^64^, ^65^ or promote tumor growth in vivo.^66^ The in vitro findings of this study demonstrate that MSC1 had largely variable outcomes compared to MSC2 and MSC3, suggesting a cell‐intrinsic response inherent to the donor beyond the cargo. MSC1 displayed relatively lower engineering efficiency (Figure 1c), lower BMP4 secretion (Figure 1d,g) and displayed reduced migration capabilities toward BTICs conditioned medium compared to MSC2 and MSC3 (Figure 2a,b). When assessing the effects of the secretome on BTIC receptor pairing and downstream signaling, MSC1 upregulated BMPR1B (Figure 4b), bypassed SMAD‐dependent downstream signaling (Figure 4d,e) and enhanced cMYC (Figure 3h). For these reasons, MSC1 was considered a deviating and underperforming donor cell source. It is unknown whether an underperforming MSC donor exhibiting sufficient anti‐BTIC capacity in vitro could perform optimally in vivo. Studies were performed to assess whether MSC1‐BMP4, despite displaying anti‐glioma properties in vitro, can replicate our previously reported therapeutic effects from an established cell line.^21^, ^22^

**FIGURE 6:**
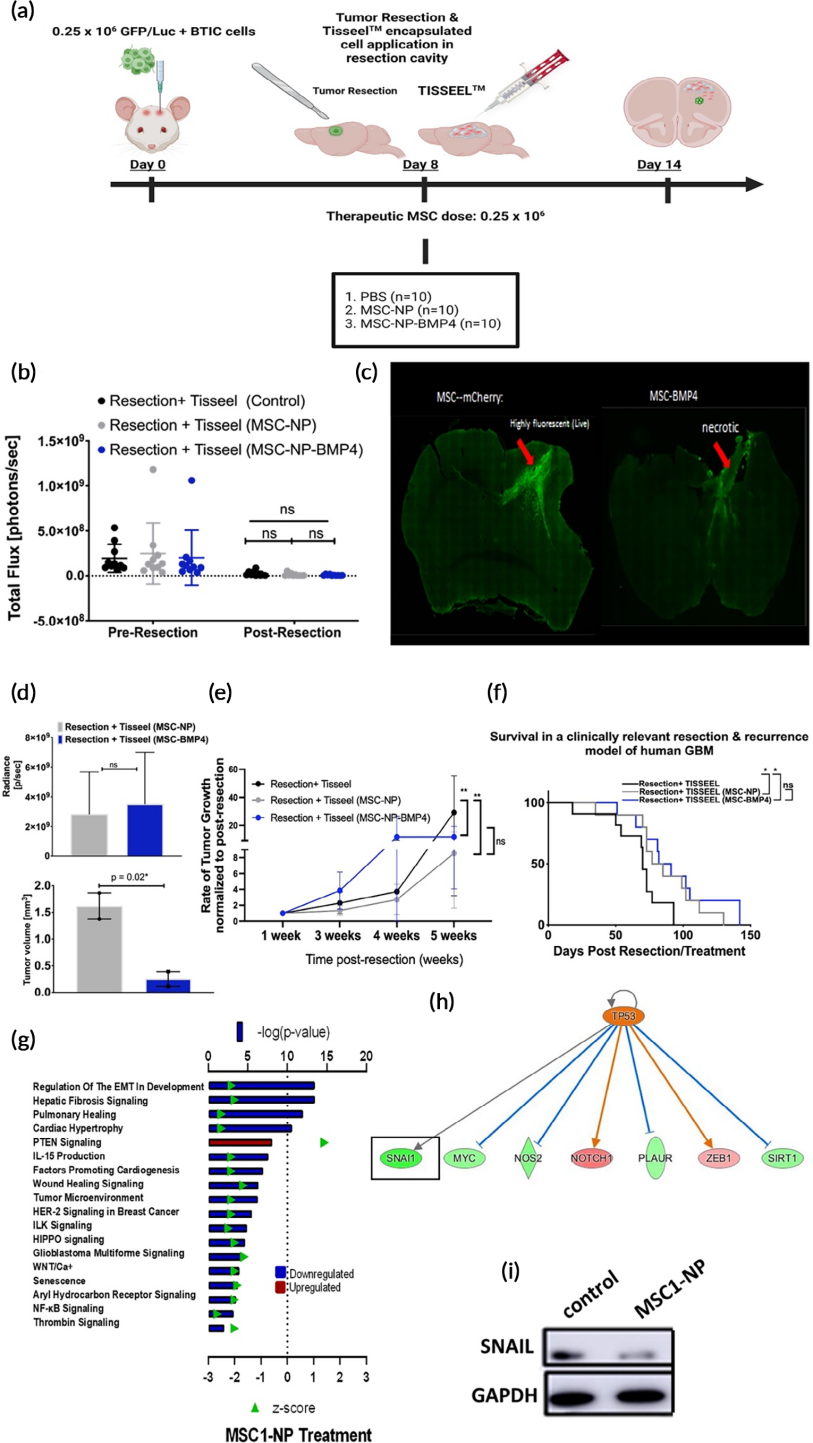
Technical success of MSC1‐BMP4 in TISSEEL is necessary, but not sufficient, for therapeutic potency in a clinically relevant resection model of human GBM. (a) Schematic representation mimicking the clinical paradigm for local implantation of nanoengineered MSC cell‐based therapy after tumor debulking. (b) IVIS image‐based measurement of tumor flux of control (*n* = 10), MSC‐NP (*n* = 10) and MSC‐BMP4 (*n* = 10) demonstrating equal resection baseline across groups, 1 day after MSC1‐TISSEEL implantation. (c) Immunofluorescence analysis of brain slices from resected animals treated with MSC‐NP and MSC‐BMP4 14‐days after implantation (*n* = 2–3). (d) Two‐week IVIS and volumetric histological analysis of mouse brains post‐treatment (*n* = 2–3). (e) Tumor growth kinetics (measured in fold change from day = 1 post‐resection). (f) Survival probability curve using log rank against control‐MSC‐NP (*p* = 0.033); MSC‐NP (*p* = 0.02); Gehan–Breslow–Wilcoxon was done to give more weight to deaths at earlier time‐points MSC‐NP (*p* = 0.06); MSC‐BMP4 (*p* = 0.04); median survival: control (70 days) versus MSC‐NP (81 days), versus MSC‐BMP4 (86.5 days); *n* = 10/group. (g) Ingenuity pathway analysis of top biological processes associated with differentially expressed cancer stem cell genes showing MSC1‐NP downregulated genes associated with mesenchymal transition. (h) Predicted MSC1‐NP activation of P53 with concomitant downstream inhibition of Snail1 (i) Immunoblot of SNAI1 of BTICs treated with MSC1 after 96 h (orange oval: activated; red oval: overexpression of activated regulator found to be P53; blue lines: leads to inhibition; orange line: leads to activation of downstream genes tested). Immunoblot confirmed SNAIL suppression consistent with downregulated EMT (IPA analysis *z* = >1.3; *p* = <0.05), *p < 0.05, **p < 0.01, and ***p < 0.001. Scale (c) 800 μm. Data represent mean ± SD (b–d); mean with 95% CI (e).

Total resection of GBM often results in residual cells left behind that display stem‐like properties and migratory characteristics.^27^, ^67^ These cells are believed to be the main culprits behind therapy resistance and relapse in GBM patients. To determine if BMP4‐engineered MSC1 can affect the overall survival of GBM‐bearing animals post‐resection, animals received human BTICs‐GFP/Luc cells injected int the cortex (Figure 6a). Tumor burden was measured using IVIS via luciferase and animals were split into groups with matched average tumor. Upon tumor confirmation at day seven, the tumor was resected and a total of 0.25×10^6^ MSC1 transfected with NPs containing mCherry or BMP4 were encapsulated in a 10 μL microbubble of TISSEEL and implanted in resection cavity. IVIS imaging revealed non‐significant residual tumor burden post‐resection among all groups, indicative of a similar starting baseline after debulking (Figure 6b). Tumor volume was assessed 2 weeks after MSC1 implantation (Figure 6c), demonstrating that MSC1‐BMP4 significantly reduced tumor size compared to MSC1‐NP controls during the early phase of recurrence (Figure 6d) consistent with the in vitro results demonstrating reduced proliferation (Figure 3c,g) and enhanced differentiation (Figure 4h,i). To assess the therapeutic impact of the underperforming MSC1 donor engineered with BMP4, animals were live imaged and tumor growth kinetics were quantified weekly after treatment. Animals were randomized for each group such that the average tumor burden across groups at baseline was non‐significant. Measurements were then taken such that each mouse served as its own control against their day‐1 treatment time‐point (post‐resection) to quantify tumor growth kinetics. MSC‐NP and MSC‐BMP4 significantly delayed recurrence by week 5 compared to untreated controls (Figure 6e), however, BMP4 engineering of MSCs did not decrease growth compared to MSCs transfected with NP.

Finally, to investigate if the underperforming MSC donor (MSC1), despite showing anti‐glioma effects in vitro, can replicate the previously reported survival outcomes of the BMP4 engineering platform compared to MSC‐NP controls,^21^ animals were evaluated for recurrence‐free survival in the first application of a resection and recurrence model of human GBM using PBAE‐based MSCs engineering. The Gehan–Breslow–Wilcoxon statistical method was used to give more weight to deaths at earlier time points, conforming to the widely acknowledged phenomenon of the short MSC retention and lifespan in vivo. MSC‐BMP4 resulted in significant survival benefits (86.6 days median survival) compared to untreated controls (70‐day median survival), however, these donor cells did not statistically improve survival compared to NP control (81‐day median survival) (Figure 6f). While MSC1‐NP extended survival to 81 days, MSC2‐NP exhibited an extended survival period of 108 days (Figure S6). Using MSC2‐NP, we further demonstrate that the donor source alone can influence survival beyond cargo activity despite a universal platform, evident from the differences in survival benefits between MSC1‐NP (mCherry) (81 days vs. control: 70) and MSC2‐NP (mCherry) (108 days vs. control: 76 days) (Figure S6). To account for overall survival without giving weight to earlier time points, the log‐rank test was performed; both MSC1‐NP and MSC1‐BMP4 imparted significant therapeutic benefits compared to untreated controls. However, outcomes failed to replicate previous reports^21^, ^22^ where BMP4 engineering outperformed one MSC line when delivered systemically or locally in a non‐resection model. Due to the minimal availability of MSC3 cells from adipose tissue derived from this donor, a survival study could not be repeated for in vivo analysis; a full assessment of MSC3 would have been optimal to understand the extent of such differences. Nonetheless, this is the first model to assess the therapeutic potential of nanoengineered MSCs using a broadly applicable NP formulation to mimic the clinical paradigm for autologous applications. Not surprisingly, these findings correlated with the in vitro results suggesting that donor intrinsic cues are predictive of downstream outcomes beyond the platform technology. Ultimately, BMP‐engineering of MSC1 failed to produce substantial survival benefits, which was predicted by aberrant in vitro screening. Such outcomes validate the need to consider the impact of donor heterogeneity to advance the selection of donors for cell‐based platforms; especially for applications intended for *autologous* cellular applications.

Given the therapeutically beneficial outcomes imparted by MSC1 (against non‐treated controls) (Figure 6), ingenuity pathway analysis (IPA) was used to assess MSC1‐specific therapeutic mechanisms by evaluating genes linked to cancer stem cells. An examination of the top regulated biological and disease functions in BTICs was retrospectively evaluated. The “regulation of epithelial to mesenchymal transition” (EMT) was the topmost downregulated process associated with the gene expression observed in BTICs upon MSC1‐NP treatment, along with the downregulation of “glioblastoma multiform signaling” pathways (Figure 6g). Furthermore, the top regulator activated was TP53, the most deregulated gene in solid cancers. GBM is frequently associated with p53 mutations which promotes processes such as EMT, self‐renewal, proliferation, and therapeutic resistance (Figure 6h). Further examination of direct correlates in stem‐cell functions regulated by p53 revealed that MSC1 inhibited cMYC, consistent with in vitro findings (Figure 4h). This is further compounded by other related genes found to be downregulated upon MSC1 treatment (SIRT1, NOS2, and SNAIL), of which, are highly implicated in GBM malignancy. Additionally, SNAIL is highly involved in EMT processes and is associated with the most aggressive subtype of GBM.^68^ To qualify if the predicted outcomes of p53 activation induced by MSC1 in BTICs downregulated SNAIL, as suggested by our pathway analysis (Figure 6h), protein expression was assessed as one surrogate indicator of p53 activation. SNAIL was found to be significantly downregulated in BTICs upon MSC1 treatment, potentially owing to the therapeutic outcomes observed with MSC1 alone and without the therapeutic cargo (Figure 6i).

Overall, donor MSC1 may not have been conducive to BMP4 engineering; nonetheless, it resulted in beneficial therapeutic outcomes and prolonged survival, albeit not significantly different than the MSC1 transfection controls. Findings demonstrate that despite MSC1 showing anti‐glioma benefits in vitro, engineering of MSC1 with BMP4 did not substantially improve survival against its counterpart suggesting that the technical capability of a donor is necessary, but not sufficient, to ensure therapeutic potency. This study validates the need to consider donor intrinsic characteristics for cellular engineering and to select optimal donors for cell therapy according to the cargo delivered and engineering platform utilized, beyond the minimum characterization requirements for MSCs as simple cellular vehicles.

## 3 DISCUSSION

In this study, we have shown that engineering or applications intended as “universal” technologies integrated into the cellular platform can offer substantial advantages if they can overcome or surpass inherent mechanisms of the donors. While developing a “universal” NP formulation is a significant goal in nanomedicine and nanotechnologies that utilize cellular applications, future technologies may work optimally when the complexity and diversity of the cellular biological systems are considered. Thus, this study suggests that despite a successful formulation meeting our cut‐off criteria across multiple donors, without compromising efficacy or safety of engineering, engineering cellular technologies continues to remain a challenge for autologous applications. By demonstrating the complex interplay between donors, engineering methods, and therapeutic cargo potency, we highlight the significance of donor‐specific characteristics in influencing outcomes, ultimately emphasizing the importance of precise donor selection for effective cellular therapies as we move into precision medicine. We demonstrate how the cellular vehicle transmits intricate signaling cues, which are inherent to the donor, that either support or contradict the expected result of the payload itself beyond the therapeutic cargo. The results of this study expand on our group's prior research by introducing a novel polymer‐based formulation, “4‐4‐6” that significantly increases BMP4 secretion (>130–1000 ng/mL) across all three donors within 48 h, demonstrating superior engineering potential over earlier lentiviral (75 ng/mL) and PBAE‐NP‐based technologies (52.5 ng).^21^, ^22^

We further demonstrate that donor‐intrinsic cues influence therapeutic outcomes despite robust cargo secretion. The current nanoplatform technology outperformed previous findings with half the cellular concentration and in a shorter amount of time across all donors, but did not statistically improve survival, indicating the need for donor screening parameters across technologies despite a robust technological platform. To our knowledge, this study is the first to examine how non‐viral nanoparticle‐mediated genetic engineering results are affected by innate MSC donor features. Our findings underline the necessity to use precision‐based strategies that consider parameters for donor selection and suitability to deliver a specific payload. Such screening approaches can identify important signaling components that may interfere with the activities of the cargo against the biological vehicle.

The selection of the “inferior” MSC1 donor illustrates the diversity of donor features given the previously established survival benefits of MSC‐BMP4.^21^, ^22^ Despite the broad and technical achievement of this platform for cellular engineering across multiple donors, the intrinsic suitability of the donor to the cargo may be more important. Furthermore, the endogenous secretome of MSCs could, in some circumstances, significantly alter the pathways that are targeted by the cargo itself. Unlike synthetic payloads, the delivery of a biological vehicle, such as MSCs, can result in antagonistic or beneficial effects of downstream cues on BTICs beyond BMP4 delivery. Our results show that, despite increased secretion from prior studies,^21^, ^22^ MSC1‐BMP4 donor did not vary from MSC‐NP control, supporting the hypothesis that the donor characteristics may hold far greater impacts on cellular nanotechnologies than previously assumed.

Furthermore, outcomes of this study builds upon previous work by introducing a new polymer‐based formulation “4‐4‐6” significantly boosting the secretion of BMP4 (>130–1000 ng/mL) across all three donors within 48 h, displaying superior engineering potential over previous lentiviral (75 ng/mL) and PBAE‐NP based engineering (52.5 ng).^21^, ^22^ To our knowledge, this study is the first to evaluate to what extent the MSC donor characteristics influence outcomes following genetic modification. Our study highlights a critical need to employ precision‐based approaches that encompass criteria for donor selection against the specified cargo, and prior to cellular engineering.

### 3.1 Downstream signaling in BTICs is a result of the combined effects of donor intrinsic cues and BMP4 delivery

This study further correlates donor source and BMP4 secretion levels to downstream effector signaling in BTICs, supporting the claim that BTIC functional outcomes may be activated by different signaling pathways imposed by the donor MSC in response to BMP4; the combination of such outcomes holds greater importance than the therapeutic cargo and the robustness of the platform technology. Therefore, it is important to note that the endogenous secretome of MSCs may, in certain cases, cause significant changes in pathways that are targeted through the engineering strategies.

Ultimately, donor profiling revealed that MSC1 is not ideal for *BMP4* engineering, which was demonstrated in screening assays (Figure 1), downstream signaling (Figure 4) and confirmed by survival studies (Figure 6) despite the broad applicability of the platform. As evidenced by MSC1 and MSC2 survival data versus controls, we demonstrate that the donor alone, irrespective of the cargo, can significantly impact therapeutic outcomes. The work demonstrates the importance of comprehensive assessments of MSCs prior to use. These considerations are especially important for pre‐clinical and IND‐enabling studies (investigational new drugs) that must account for differences in cell sources as well as changes in culture environments during scale up and manufacturing for autologous applications.

Although MSCs offer versatility for both autologous and allogeneic therapy, a key limitation of this study lies in the absence of an immune system in the animal models. While significant differences in the MSCs' engineering potential were revealed, future investigations can leverage these findings using an immunocompetent background. It is also of note to highlight alternative approaches to resection models where non‐ablative and multi‐functional devices can be used to enable preservation of tissue for in depth analysis of the stroma post‐implantation.^69^ Given the findings of this work, future studies using an immunocompetent model can enable an in‐depth examination of this therapy's efficacy alongside standard treatments like radiation and chemotherapy. Immunocompetent or humanized animal models hold clinical relevance and can shed some light on how immunological interactions can impact donor‐specific mechanisms in a more physiologically meaningful context. To achieve a more precise representation, the investigation of the immunological response post‐implantation becomes pivotal, and will allow for a thorough assessment of safety, efficacy, and potential donor‐specific factors influencing therapeutic potency or limitations.

Furthermore, strategies utilizing plasmid technologies continue to be explored for clinical applications for GBM (NCT05698199) and others. PBAE nanoparticles can be used to incorporate a range of nucleic acids, including DNA, mRNA, and/or siRNA with similar intracellular delivery properties.^70^, ^71^, ^72^, ^73^, ^74^, ^75^ Nonetheless, alternative technologies that can optimally incorporate surface receptors or more advanced means that can override the donor‐intrinsic mechanisms may prove more beneficial.

### 3.2 Process refinement

Healthcare is becoming increasingly reliant on cell and gene therapy for a wide array of indications; thus, ensuring the reproducibility of future studies for autologous applications using the same donor MSC line hinges on the meticulous control over transfection protocols and processing methods during expansion and engineering. Several factors may be considered—(1) Characterization of donor MSC: MSCs should be assessed for morphology, preservation of mesodermal differentiation and multipotency (function), and expression of surface receptors through flow cytometry (identity) against non‐modified MSCs from the same donor. (2) Engineering process: efforts are directed toward observation of transfection efficiency, high degree of quality control in the manufacturing process of NP‐engineered MSCs, release kinetics and cut‐off criteria (e.g., release of 10–100 ng BMP4) compared to non‐modified MSCs. (3) Isolation process, storage, and handling: to enhance precision, all MSCs isolated from tissue should be cryopreserved before engineering or modification. Experimental groups can be randomized then assigned to experimental groups and replicates within the same passage and during the same timeframe of the engineered group (within hours). (4) Validation: Changes or alterations in proliferation rate, viability or intracellular pathways after engineering are assessed against non‐engineered cells. Notwithstanding process control and refinement for each donor, the significant heterogeneity of MSCs across donors for “off‐the‐shelf” therapy necessitates contingency plans that can navigate the complexities of biomanufacturing. One such approach may include the development of alternative NP formulations that can serve as readily available backups when needed. Another strategy can focus on the development of modular formulations tailored based on individual donor characteristics. Such biological features inherent to the MSC donor line can act as surrogate indicators to guide the selection of the most optimal “off‐the‐shelf NP formulation for clinical applications”.

## 4 CONCLUSION

Overall, this study demonstrates that potency of biological vehicles for therapy are not “donor‐agnostic”. Interactions when combining a cellular vehicle (MSCs), a broadly applicable engineering technology (NPs), and therapeutic cargo (BMP4), may override the intended effects of therapeutic cargo alone, such that the combined effects can go beyond the presumed cargo function due to the biological vehicle. Findings support the claim that despite successful and robust technical capabilities of PBAE‐NPs, therapeutic potential is dependent on the combined effects of the success of the genetic engineering itself as well as intrinsic characteristics of the donors' suitability to the cargo. We show here that despite robust technical capabilities of the NP platform, donor intrinsic cues hold greater influence on therapeutic outcomes for cell therapy.

## 5 MATERIALS AND METHODS

### 5.1 Primary patient derived cells

All MSCs (MSC1, MSC2, and MSC3) used in this study were derived from adipose tissue from patients undergoing transsphenoidal surgery at the Mayo Clinic following Mayo Clinic IRB approved protocol; MSCs were extracted and isolated from adipose tissue from the right lower quadrant of the abdominal fat pad (in between the umbilical plane and the right inguinal ligament), under the skin of patients undergoing transsphenoidal surgery for a benign tumor resection and where this fat is utilized to reconstruct the skull base defects post‐surgery. The source of fat is far away from the surgical site of the primary tumor resection incision and the left‐over adipose tissue was sent to lab where MSCs were isolated. MSCs were grown in complete medium, which consists of advanced minimal essential medium (MEM) (Gibco 12492013), 5% PLTMax (MilliporeSigma), 1% GlutaMAX (Life Technologies), and 0.2% heparin sodium injection (NDC 63323‐540‐57). MSC base medium (without heparin) was used for transfection.

GBM120 and GBM1A were used in this study, herein termed as BTICs are both GBM patient‐derived glioblastoma cell lines. Cell line GBM1A was derived by Galli et al.^59^ and cell line GBM120 was derived from tissue obtained intraoperatively as described by our team.^76^ Some experimental protocols required the use of GBM base media (media without EGF/FGF mitogens).

### 5.2 MSC transfection

For transfection of MSCs, PBAE nanoparticle was used. The detailed procedures of PBAE polymer synthesis and screening of PBAE polymer analogues for transfection is provided in Appendix S1. Based on the preliminary screening, we found PBAE 4‐4‐6 (Figure S1) as our leading polymer and hence all the following studies were performed using PBAE 4‐4‐6 polymer‐based nanoparticle. Passage four adipose‐derived MSCs were seeded at a density of 10^4^ cells/cm^2^ and cultured in MSC complete medium, as described above. At 70%–80% confluency, complete media was removed, cells were washed 1× with phosphate buffered saline (PBS) and replaced with base medium (0.2 mL/cm^2^ surface area). PBAE nanoparticles (NPs) with plasmid DNA encoding mCherry or BMP4 were formulated according to a modification of previously described procedures.^21^ Briefly, PBAE 4‐4‐6 and plasmid DNA were dissolved separately in 25 mM sodium acetate buffer (pH 5), then mixed for self‐assembly into NPs at a PBAE‐to‐DNA mass ratio of 70:1, with a DNA concentration in the NPs 22.5 μg/mL. To the NPs was added an excipient solution of sterile sucrose (75 mg/mL) and magnesium chloride (MgCl_2_, 50 mM) at a 2:3 (v/v) ratio of excipient solution to NPs. The NPs with excipient were then frozen at −80°C and lyophilized for 24 h, then stored dry at −20°C or below with desiccant until use. Lyophilized NP‐cherry or NP‐BMP4 were reconstituted in sterile water to a final DNA concentration of 22.5 μg/mL and added to each flask (40 μL NPs or 900 ng DNA/cm^2^ surface area), and the cells were incubated at 37°C and 5% CO_2_ for 1.5 h. Transfection media was discarded, and cells were washed 1× with PBS before complete MSC medium was added. Cells were left to recover for 12–24 h before they were detached from the flask and re‐plated for experimental assays or media collection.

### 5.3 Collection of conditioned media

Transfected or naïve adipose‐derived MSCs were seeded in six‐well plates at a density of 2.5 × 10^5^ cells/well and grown in MSC complete media at 37°C and 5% CO_2_. After 24 h, cells were washed with PBS, and 2 mL of GBM base media was added per well for conditioning; base was used to collect MSC secretome without the influence of mitogens otherwise found in ‘complete’ GBM media. Cells were incubated at 37°C and 5% CO_2_ for 48 h, then media was collected from each well, centrifuged at 300 × *g* for 5 min to remove debris, and frozen at −80°C for future use.

### 5.4 BMP4 secretion

Primary adipose‐derived MSCs were transfected with mCherry‐NP or BMP4‐NP following above‐described method. Each group was seeded in T75 flask at a density of 5 × 10^5^ cells/10 mL and grown in MSC complete medium or 5 × 10^5^/2 mL in 6‐wells in GBM‐based medium at 37°C and 5% CO_2_. Media was collected and BMP4 secretion measured using the BMP‐4 Human ELISA kit from Thermo Fisher Scientific according to manufacturer recommendations (Cat #EHBMP4). Secretion of BMP4 was also measured in GBM‐based medium following the described methods for GBM‐base media above. MSC groups for each donor were seeded in 6‐well plate at a density of 5 × 10^5^ cells/2 mL and BMP4 secretion was measured using the BMP‐4 Human ELISA kit from Thermo Fisher Scientific according to manufacturer recommendations (Cat #EHBMP4).

### 5.5 Cytokine secretion

Human cytokine array proteome profiler (R&D ARY005B) was used to determine relative levels of a selected number of cytokines and chemokines according to manufacturer protocol. Engineered MSCs were plated in six‐well plate at a concentration of 0.5 × 10^6^/2 mL/well for 24 h. Conditioned media was collected, spun down and filtered through a 0.45 μm filter and frozen until day of analysis. Filtered media was spotted in duplicates on a membrane and intensity analyzed through ImageJ software.

### 5.6 Transwell migration

The effect of nanoparticle uptake on MSC migration toward BTIC‐conditioned medium was assessed in vitro using Costar transwell assays (8 μm pore size). Non‐transfected MSCs (naïve), MSC‐NP (transfection control, with mCherry plasmid), and MSC‐NP‐BMP4 (engineered MSCs) were tested 48 h after transfection. Cells were plated in the upper chamber of a 24‐transwell plate at a density of 4 × 10^4^ cells/well with 0.5% fetal bovine serum (FBS); control medium or GBM‐conditioned media supplemented with 5% FBS was placed in the bottom transwell chamber. After 24 h of incubation, membranes were fixed and nuclear staining was utilized for counts.

### 5.7 Cell viability

Viability of nanoparticle transfected MSCs was evaluated using a (3‐(4,5‐dimethylthiazol‐2‐yl)‐2,5‐diphenyltetrazolium bromide) (MTT) assay. Non‐transfected MSCs and mCherry/NP‐MSCs were plated in a 96‐well plate at a density of 3 × 10^3^ cells/well and incubated for 7 days (media was changed every 3 days). After incubation, the culture medium was aspirated and replaced with 100 μL/well of 10% MTS in phenol red free DMEM and incubated at 37°C for 4 h. After 4 h, 75 μL of the MTT/DMEM/DMSO media was removed and replaced with phenol free DMEM. Absorbance value at 570 nm was measured using a plate reader.

### 5.8 RNA collection

GBM cells (2 × 10^4^/cm^2^) were plated T25 flasks coated with mouse laminin I (Cultrex) with GBM complete medium. After 24 h, cells were treated with a 1:1 ratio of MSC conditioned medium:GBM complete medium or GBM base medium:GBM complete medium. After 48 or 96 h of treatment, cells were washed with PBS, dissociated with 1 mL TRIzol reagent (Ambion). RNeasy Mini Kit (Qiagen) was used to isolate the RNA according to manufacturer instructions.

### 5.9 Immunocytochemistry

Eight‐chamber slides (Lab‐Tek II Chamber Slide System) were coated with poly‐L‐ornithine solution (Sigma) at room temperature for 30 min, washed then coated with laminin for 2 h in 37°C and 5% CO_2_. GBM cells were then plated at a density of 1 × 10^4^ cells/well in GBM complete media. After 48 h, GBM cells were treated with a 1:1 ratio of MSC conditioned media to GBM complete media or GBM base media to GBM complete media and incubated for 10–14, with media replaced after 5 days. Images collected with Zeiss Confocal LSM800 microscope.

### 5.10 Protein collection and Western blotting

GBM cells (2 × 10^4^/cm^2^) were seeded in laminin coated T25 flasks and cultured in GBM complete medium. After 24 h, the complete medium was removed, the cells were washed with PBS, and treated with a 1:1 ratio of MSC‐conditioned media and BTIC complete media or GBM base media and BTIC complete media. After 96 h, cells were washed with cold PBS, and 100 μL of phosphatase inhibitor was added to. Protein was quantified using the BCA Protein Assay kit according to manufacturer's instructions. Antibodies were used according to manufacturer: SMAD 1/5/8 (Cell Signaling; 12430S), STAT3 (Cell Signaling; 9131S), pERK1/2 (Cell Signaling; 9101S), MAPK (Cell Signaling 9102S), GFAP (Dako Z0334), P27 (Cell Signaling; 3686S), and p21 (Cell Signaling; 3685).

### 5.11 TISSEEL fibrin sealant application

The product consists of two identical syringes filled with thrombin (1 mL) + fibrinogen (1 mL) using Fibrinogen and Thrombin preparations from TISSEEL Fibrin Sealant (Baxter). TISSEEL (approved for clinical by the US Food and Drug Administration, STN 103980) is widely used in neurosurgery to achieve hemostasis. The applicator is designed to allow simultaneous ejection from the syringes using a dual‐set plunger. A Y‐piece (provided with the product) acts as a joint piece to the male connectors at the end of each syringe barrel for simultaneous mixture and dispensing. The final applicator tip is attached to the Y‐piece and joined via a luer‐lock mechanism that allows mixture and equal dispensing of product components consisting of (fibrinogen, thrombin, and cell mixture at a 2:1:1 ratio, respectively).

### 5.12 Seeding MSCs in TISSEEL

TISSEEL (approved for clinical by the US Food and Drug Administration, STN 103980) is widely used in neurosurgery to achieve hemostasis. To prepare MSC‐encapsulation in TISSEEL, microbubbles were created under sterile conditions using fibrinogen and thrombin preparations from TISSEEL Fibrin Sealant (Baxter). A concentration of 2.5 × 10^4^ cells/2 μL of cell suspension in PBS was diluted in 2 μL of LRS and mixed with 2 μL of thrombin (400–625 units/mL)—gelation was induced by physically mixing the cell suspension with 4 μL of fibrinogen (67–106 mg/mL).

### 5.13 Time‐lapse imaging

A microbubble composed of 2.5 × 10^5^ MSCs/10 μL of TISSEEL mixture was added to a six‐well and allowed to solidify for 15 min prior to adding culture conditions containing serum or BTIC conditioned medium. The six‐well was in the live‐imaging system (ZEN) and allowed to equilibrate. Fluorescent images under mCherry channel were captured at 10× magnification every 20 min for 48–96 h in eight different locations per well.

### 5.14 Tumor implantation and resection

Animal experiments were conducted following the guidelines of the Institutional Animal Care and Use Committee (IACUC) at Mayo Clinic (protocol—A00004347‐19). Six‐ to 8‐week‐old athymic nu/nu male mice received intracranial tumors through stereotactic injection of 0.25 × 10^6^ luciferase‐bearing GBM1A‐green fluorescent protein after a midline incision in the right cortex in the following coordinates with respect to the bregma: 1.5 mm lateral, 1.34 mm anterior, and 1.8 mm. Tumor burden was measured using in vivo Imaging System (IVIS) via luciferase (Perkin Elmer) and animals were split into groups with matched average tumor burden among the groups before resection and treatment. Upon tumor confirmation at day 7, a 3 mm diameter biopsy punch was used to outline the resection surface from the original tumor coordinates and tumor tissue was carefully resected using curved micro‐scissors at a depth of 1.5 mm (leaving 0.3 mm of tumor to mimic recurrence) as previously reported.^77^, ^78^ To establish uniform and consistent burden across all groups, tumor implantation was conducted blindly across all subjects and prior to the selection of study groups. Upon tumor growth using IVIS, subjects were then randomly shuffled and assigned using Excel and GraphPad such that the average of tumor flux between groups is equivalent with no statistical difference. This ensures that all comparative groups commenced the study with an equivalent tumor burden before treatment initiation. To further reduce bias, the prep and encapsulation of MSC‐TISSEEL during surgery was performed by another member not involved in the resection and implantation (surgical) procedures. Further, animals with no tumor‐growth were excluded from study as they do not represent a clinical model of GBM. Similarly, animals with no tumor recurrence after MSC implantation were excluded to prevent the possibility of assigning minimal or absent tumors to treatment groups, thereby avoiding potentially misleading positive outcomes that may be inaccurately assigned to the MSC therapy. A microbubble composed of 0.25 × 10^6^ MSCs/10 μL of TISSEEL (2 μL cells:4 μL fibrin:4 μL thrombin) was implanted in the surgical cavity. For survival analysis, 10 mice per group were used. For distribution and tumor volumetric assessment, three mice per group were sacrificed 2 weeks after injection.

### 5.15 Statistical analysis

Statistical analysis was performed using GraphPad Prism 9. Data distribution was determined by ANOVA with Tukey's or Bonferroni (*p* value adjusted) multiple comparison post‐hoc analysis; blot comparisons were corrected for multiple comparisons by controlling for false discovery rate (FDR) where appropriate. Kaplan–Meier reporting median survival times with a 95% confidence interval and analyzed by Gehan–Breslow–Wilcoxon test to assess impact at earlier timepoints after resection and treatment, and log‐rank for overall survival. The effect size was evaluated in accordance with our previously published studies of the PDX mouse GBM tumor model for mouse brains.^22^, ^77^ Results represent deviation (SD) unless otherwise noted. Statistical significance is represented by *p < 0.05, **p < 0.01, ***p < 0.001, and ****p < 0.0001 compared to the control. #*p* < 0.05, ##*p* < 0.01, ###*p* < 0.001, and ####*p* < 0.0001 compared to the transfection control (NP) or $*p* < 0.05, $$*p* < 0.01, $$$*p* < 0.001, and $$$$*p* < 0.0001 compared to recombinant human BMP4 (rhBMP4).

### 5.16 Animal inclusion statement

Glioblastoma (GBM) occurs more frequently in males than females, with a ratio of 1.6:1. Addressing sex‐specific differences in GBM pathology and treatment responses will require future research encompassing both male and female animal models. To isolate the extent of MSC donor variability, previous studies forming the foundation of this nanotechnology (MSC‐PBAE‐NP) have predominantly concentrated on investigating male animals. Thus, male‐only animals were used to maintain consistency and minimize unpredictability, allowing us to isolate the extent of MSC donor variability more closely.^22^ Using male animals enabled a more targeted investigation to build on our previously published findings,^21^, ^22^ while acknowledging the limitations in extrapolating these results to female patients.^79^ Addressing gender‐specific differences in GBM pathology and therapeutic responses will allow more specific parameters for donor MSC selection.^79^ Animal randomization was done after intracranial injection and post‐resection^77^, ^78^ to validate recurrence and minimize inclusion of non‐recurrent animals which would result in a potential false‐positive of our treatment groups.

## AUTHOR CONTRIBUTIONS

**Rawan Al‐Kharboosh:** Conceptualization; data curation; formal analysis; investigation; methodology; writing – original draft; writing – review and editing. **Alex Bechtle:** Data curation; writing – original draft; writing – review and editing. **Stephany Y. Tzeng:** Formal analysis; investigation; resources; writing – original draft; writing – review and editing. **Jiaying Zheng:** Formal analysis; writing – review and editing. **Sujan Kumar Mondal:** Writing – review and editing. **David R. Wilson:** Investigation; writing – review and editing. **Carlos Perez‐Vega:** Visualization; writing – review and editing. **Jordan J. Green:** Funding acquisition; investigation; supervision; writing – review and editing. **Alfredo Quiñones‐Hinojosa:** Funding acquisition; investigation; supervision; writing – review and editing.

## FUNDING INFORMATION

National Institutes of Health, R01CA195503 (A. Q. H., J. J. G.), National Institutes of Health P41EB028239 (J. J. G.)., and Maryland Technology Development Initiative (TEDCO) ‐ Maryland Innovation Initiative (MII) (J. J. G.).

## CONFLICT OF INTEREST STATEMENT

J. J. G. is a co‐founder, manager, and CTO of Dome Therapeutics. A. Q. H. is a co‐founder, manager, and CMO of Dome Therapeutics. J. J. G., S. Y. T., D. R. W., and A. Q. H. are a co‐inventor on patents related to the manuscript. A. Q. H. is principal investigator and R. A. co‐investigator of IND application for phase I non‐randomized trial evaluating safety and efficacy of MSCs for GBM. R. A. is co‐founder of AtPoint. Any potential conflicts of interests are managed by Mayo Clinic and the Johns Hopkins Committee on Outside Interests.

## Supporting information


**APPENDIX S1:** Supporting information.^80^, ^81^, ^82^, ^83^

**VIDEO S1.** MSC‐cherry encapsulated in TISSEEL are viable and migrate out of TISSEEL gel encapsulation. MSC‐cherry and imaged via time‐lapse microscopy for a period of 48–96 h at 20×.

## Data Availability

Data needed to evaluate the conclusions of the paper are present in the paper or the supplementary materials. Additional data related to this paper may be requested from the authors.
