# Supplementary material for: Therapeutic potential and impact of nanoengineered patient‐derived mesenchymal stem cells in a murine resection and recurrence model of human glioblastoma
Source: Bioeng Transl Med. 2024 May 7;9(6):e10675. doi: 10.1002/btm2.10675 (PMC11558202; doi:10.1002/btm2.10675)
Supplement: Supplementary file 1 — APPENDIX S1: Supporting information. 80 , 81 , 82 , 83 [file BTM2-9-e10675-s001.docx]

Supplementary Materials for

**Therapeutic Potential and Impact of Nanoengineered Patient-derived Mesenchymal Stem Cells in a Murine Resection and Recurrence Model of Human Glioblastoma**

Rawan Al-kharboosh, Alex Bechtle, Stephany Y. Tzeng, David R. Wilson, Jiaying Zheng, Sujan Kumar Mondal, Carlos Perez-Vega, Jordan J. Green, Alfredo Quiñones-Hinojosa*

*Corresponding author: Quinones-Hinojosa.Alfredo@mayo.edu

**This PDF file includes:**

Supplementary Text

Figs. S1 to S6

Tables S1

Movies S1

References

**Other Supplementary Materials for this manuscript include the following:**

Movies S1

Supplementary Text

*Polymer synthesis for high-throughput transfection screening*

For high-throughput screening, polymers were synthesized using previously reported protocols (*1*) using the monomers in **Table S1**. Briefly, one diacrylate backbone monomer ("B") and one side-chain monomer ("S") were mixed at a B-to-S molar ratio of 1.1:1 and reacted with stirring for 24 h at 85°C. These acrylate-terminated base polymers were then dissolved in anhydrous dimethyl formamide (DMF) at 200 mg/mL, precipitated twice in diethyl ether to remove unreacted monomer, and then dried under vacuum for 24 hr to remove excess ether. The resulting base polymer was weighed, dissolved in anhydrous dimethyl sulfoxide (DMSO) at 200 mg/mL, and dried again under vacuum for 24 hr before being stored at -20°C with desiccant until use. For final end-capped PBAE synthesis, the base polymers were aliquoted into 384-well plates. To each backbone was added a solution of end-cap monomer in anhydrous DMSO for a final concentration of 100 mg/mL base polymer and 0.1 M end-cap monomer. These were allowed to react with shaking for 2 hr at room temperature, then diluted to 50 mg/mL in DMSO and aliquoted to 384-well plates (5 µL/well). These plates were frozen at -20°C and stored with desiccant until use. Polymers are named according to their component monomers, e.g., "B4-S4-E6," or "4-4-6" for brevity.

For transfection, MSCs were seeded into 384-well plates at 10^4^ cells/cm^2^ in complete growth medium and allowed to adhere overnight. The next day, the medium was replaced with 50 µL/well basal medium. Nanoparticles (NPs) were formed by diluting the PBAEs in 384-well plates with 25 mM sodium acetate buffer (pH 5, NaOAc) and mixing them via pipetting with DNA diluted in NaOAc at PBAE-to-DNA mass ratios of 40 w/w and 80 w/w. NPs were added to the cells in 5 µL volume for a final dose of 40 ng DNA/well. The cells were incubated with particles for 2 hr at 37°C and 5% CO_2_, and then the medium was changed to 50 µL fresh complete growth medium. After 48 hr, transfection was assessed by fluorescence imaging using a Cellomics ArrayScan VTI (Thermo Fisher) as previously described (*1*)^,^(*2*).

*Polymer synthesis for transfection optimization*

The leading PBAE from the high-throughput screen, polymer 4-4-6 **(Figure S1)**, was resynthesized at larger scale and greater purity for further study following previously reported protocols.(*3*) The base polymer B4-S4 was synthesized neat at a 1.1:1 molar ratio (B:S) at 85°C with stirring for 24 hr. This base polymer was dissolved in anhydrous tetrahydrofuran (THF) and reacted with 10-fold molar excess of the end-cap E6 for 2 hr at room temperature. The polymer was purified by precipitation in diethyl ether followed by two washes in diethyl ether, then dried under vacuum for 48 hr. The final end-capped PBAE was dissolved in anhydrous DMSO at 100 mg/mL and stored at -20°C with desiccant in small aliquots to minimize freeze-thaw.

Optimization was completed in two steps. First, MSCs were seeded in 96-well plates at 10^4^ cells/cm^2^ in complete growth medium and allowed to adhere overnight. The next day, the media was replaced with 100 µL/well of basal medium. NPs were formed by diluting both DNA and PBAE 4-4-6 in NaOAc and mixing at PBAE:DNA mass ratios of 30, 50, and 70 w/w. NPs were added to the cells in 20 µL volume (300 ng DNA/well) and allowed to incubate for 1.5 hr at 37°C with 5% CO_2_. The media and NPs were replaced with fresh complete MSC medium, and cells were analyzed by flow cytometry after 48 hr.

After selection of the best NP formulation (4-4-6, 70 w/w), the lyophilized NP formulation was optimized based on previously reported work showing that PBAE/DNA NPs can be lyophilized with 30 mg/mL sucrose without losing efficacy (*2*)^,^ (*4*). NPs were formulated from DNA and PBAE 4-4-6 as described at varying concentrations, then mixed with lyoprotectant excipients sucrose and magnesium chloride (MgCl_2_) for final concentrations of 30 mg/mL sucrose and a range of concentrations from 0 to 200 mM of MgCl_2_. The NPs with excipients were frozen at -80°C and lyophilized for 24 hr, then stored dry at -20°C with desiccant until use. Before transfection, MSCs were seeded in 24-well plates at 10^4^ cells/cm^2^ and allowed to adhere overnight. The next day, growth medium was replaced with 500 µL/well basal medium. The NPs were reconstituted in water and added to the cells in 100 µL volume at doses ranging from 526.5 ng/well to 3.75 µg/well DNA. After 1.5 hr of incubation, the medium was replaced with complete growth medium, and the cells were analyzed by flow cytometry after 48 hr. The leading formulation (4-4-6, 70 w/w, lyophilized with 30 mg/mL sucrose and 20 mM MgCl_2_) was used for all subsequent experiments.


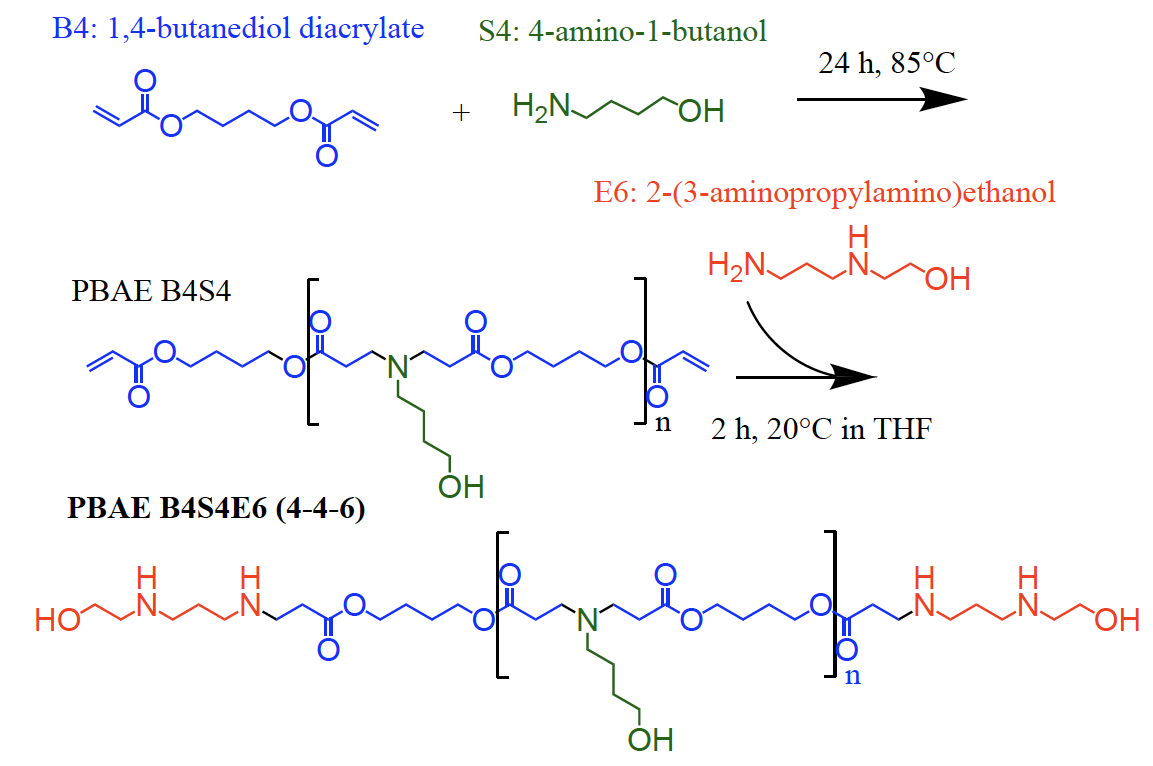


Figure S1. Synthesis scheme and chemical structure of PBAE B4S4E6 (also abbreviated PBAE 4-4-6)


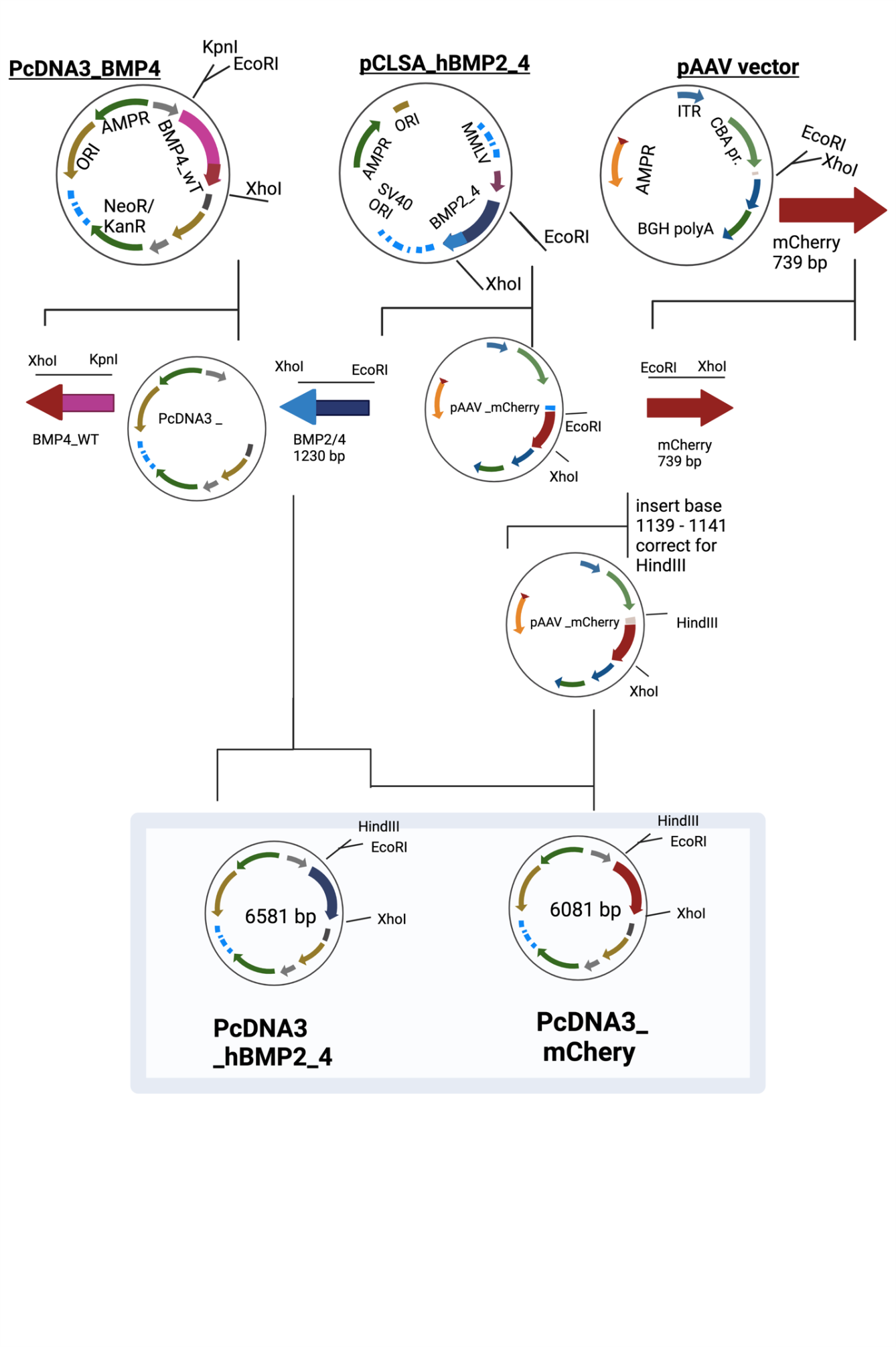


Figure S2. Design of plasmid vector. pcDNA_BMP4, and pcDNA_mCherry delivered by nanoparticles of the same backbone and under the same promoter.

****Figure S3. ***SDF-1 secretion****.* ***(A)*** Secretion levels of SDF-1 between MSC1-NP versus MSC1-BMP4 from conditioned medium at 48-hours measured through human cytokine array. **(B)** Secretion levels of SDF-1 between MSC2-NP versus MSC2-BMP4 from conditioned medium at 48-hours measured through cytokine array analysis **(C)** Schematic representation of transwell migration. Data represent mean ± standard deviation (SD) of three independent experiments pooled and replicated at least twice or more. *, p < 0.05 compared to the transfection control (NP) under the same conditions. ns, not significant. BTICs, brain tumor-initiating cells; CM, conditioned medium; control, MSC medium; GBM, glioblastoma; SDF-1, stromal cell-derived factor 1.


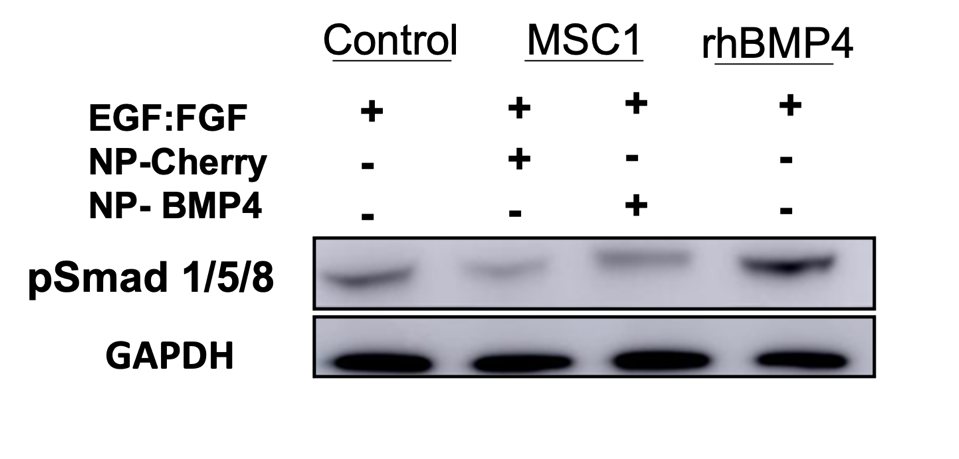


Figure S4. Immunoblot of p-smad1/5/8 in patient-derived BTICs. Confirmation of widely acknowledged rhBMP4-induced SMAD signaling in human-derived BTICs. MSC1 bypasses SMAD, despite BMP4-engineering consistent with *in vitro data*.


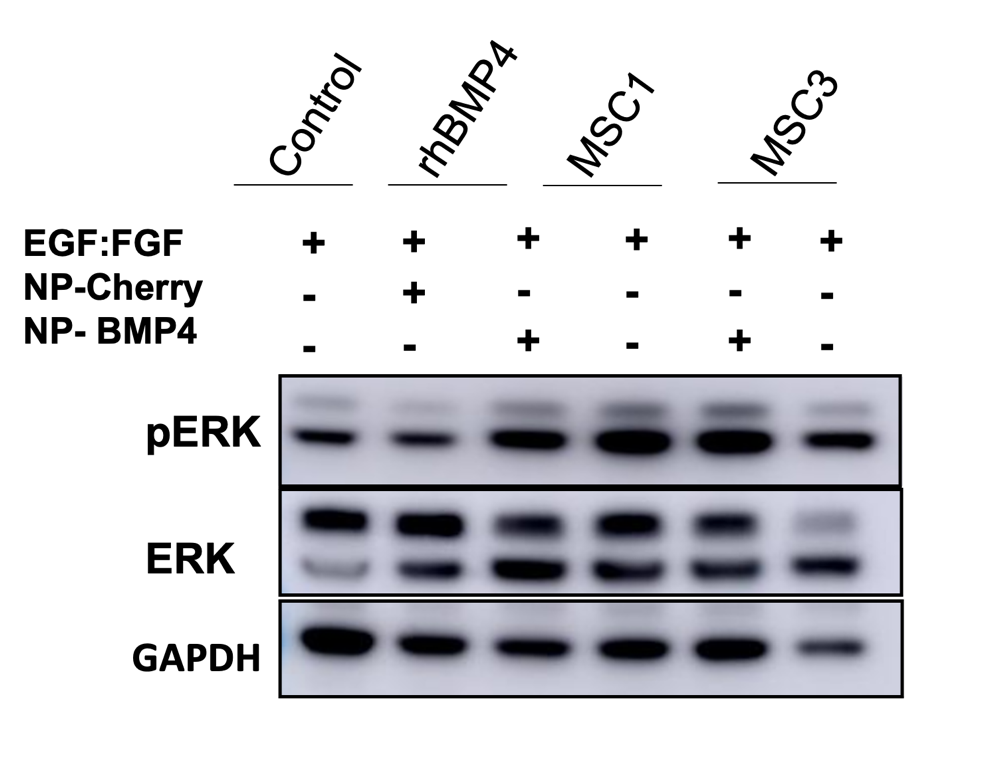


Figure S5. Immunoblot of ERK in patient-derived BTICs. ERK inactivation in the presence of rhBMP4 compared to MSC1 (low secreting) and MSC 3 (highest secreting) donors. MSC donors inherently induce ERK activation and BMP4 engineering reduces this signaling pathway consistent with rhBMP4 alone due to preferential SMAD; this is not the case for the low secreting MSC1 which enhances SMAD-independent pathway despite BMP4 engineering consistent with *in vitro* findings.

Figure S6: TISSEEL encapsulated MSC2. (A) Real-time tumor burden pre- and post-resection. (B) Survival curve log rank (mantel-cox) p = 0.0049**; Gehan-BreslowWilcoxon p = 0.01*; median survival: control (76 days) Vs. AMSCs (108 days); Ratio 0.7037. Hazard ratio (logrank): 3.771; 95 CI of ratio 0.939 to 15.14. Control (n=6) and MSC2-NP (mCherry) (n=6)l; Data represent mean ± standard deviation (SD) for (A).

Table S1. Constituent monomers for synthesis and screening of PBAE library.

Movie S1. MSC-cherry encapsulated in TISSEEL are viable and migrate out of TISSEEL gel encapsulation. MSC-cherry and imaged via time-lapse microscopy for a period of 48 -96hrs at 20x.

1. B. Mishra *et al.*, A combinatorial library of biodegradable polyesters enables non-viral gene delivery to post-mitotic human stem cell-derived polarized RPE monolayers. *Regen Eng Transl Med* **6**, 273-285 (2019).

2. S. Y. Tzeng *et al.*, Non-viral gene delivery nanoparticles based on poly(beta-amino esters) for treatment of glioblastoma. *Biomaterials* **32**, 5402-5410 (2011).

3. L. J. H. S. Y. Tzeng, M. G. Pomper, J. J. Green, biomaterial-mediated cancer-specific DNA delivery to liver cell cultures using synthetic poly(beta-amino ester)s. *J Biomed Mater Res A* **101**, (2013).

4. H. Guerrero-Cazares *et al.*, Biodegradable polymeric nanoparticles show high efficacy and specificity at DNA delivery to human glioblastoma in vitro and in vivo. *ACS Nano* **8**, 5141-5153 (2014).
